# Jamestown Canyon Virus Rapidly Adapts to Mosquito Cells Through Multiple M Segment Mutations

**DOI:** 10.3390/v18091018

**Published:** 2026-09-15

**Authors:** Sarah Dysinger, Tamanna Srivastava, Sara Cherry, Paul Bates

**Affiliations:** 1 Department of Microbiology, Perelman School of Medicine, University of Pennsylvania, Philadelphia, PA 19104, USA; 2 Department of Pathology and Laboratory Medicine, Perelman School of Medicine, University of Pennsylvania, Philadelphia, PA 19104, USA

**Keywords:** Jamestown Canyon virus, orthobunyavirus, arbovirus, insect cell adaptation, California serogroup viruses, reverse genetics

## Abstract

Jamestown Canyon virus (JCV) is a mosquito-borne orthobunyavirus with a broad host and vector range. Despite increasing mosquito-to-human spillover, the viral determinants governing host adaptation remain poorly defined. We examined changes in JCV replication during serial passage in mosquito cells and linked adaptive changes in viral fitness to specific genetic mutations. In mosquito-derived C6/36 cells, JCV exhibited a distinct “lag-burst” phenotype in which viral replication remained nearly undetectable for 10 days before abruptly increasing. Following reinfection of fresh C6/36 cells, JCV passaged once in mosquito cells exhibited robust replication with no detectable lag phase. Sequencing identified seven substitutions compared to the published reference sequence, including S193G in Gn, three NSm substitutions (P346A, M352V, and W371R), and three Gc substitutions (H890Y, I1365V, and L1394F). Using a plasmid-based reverse genetics system, individual mutations were introduced into recombinant JCV and tested for effects on mosquito cell replication. All tested mutations independently enhanced replication efficiency, demonstrating that adaptation can arise through multiple independent genetic pathways. However, no individual mutation fully reproduced the phenotype acquired naturally through mosquito cell passage. Together, these findings demonstrate that JCV rapidly adapts to mosquito cells under minimal selective pressure and highlight the potential for emergence of increasingly well-adapted viral variants.

## 1. Introduction

Jamestown Canyon virus (JCV), a mosquito-borne orthobunyavirus in the family *Peribunyaviridae*, was originally isolated from mosquitoes in 1961 in Jamestown, Colorado [1,2]. Subsequently, JCV was found to be widely distributed across the northern United States and Canada [3,4]. As an arbovirus, JCV circulates between mosquito vectors and vertebrate hosts, with white-tailed deer serving as the primary amplification host [3,5,6]. However, evidence of infection has also been documented in numerous incidental hosts, including caribou, dogs, cattle, horses, foxes, and polar bears, among others [7,8,9]. Serological studies indicate that exposure is common in both reservoir and incidental hosts, with regional seroprevalence in deer exceeding 80%. Up to 50% of humans in endemic areas show evidence of prior exposure, but humans are considered dead-end hosts [10]. Unlike other arboviruses that are restricted to one or two competent mosquito vectors, JCV has been detected in at least 46 mosquito species spanning multiple genera, and several species are thought to sustain year-round transmission [11,12,13,14,15,16,17,18,19]. Together, these observations point to a virus that is both ecologically widespread and highly promiscuous in its host associations. Despite this, JCV remains comparatively understudied, even as expanding vector ranges and documented reassortment among orthobunyaviruses raise concern for the emergence of novel variants [14,20,21,22,23].

Underlying this broad host range is a compact, tri-segmented RNA genome that supports replication in both mosquito vectors and mammalian hosts. JCV possesses a single-stranded, negative-sense RNA genome consisting of the small (S), medium (M), and large (L) segments. The S segment encodes the nucleocapsid (N) protein and the nonstructural protein NSs [24]. Based largely on studies of La Crosse and Bunyamwera viruses, NSs is believed to modulate host immune responses to promote infection in both mammals and mosquitoes [25,26]. The L segment encodes the viral RNA-dependent RNA polymerase. The M segment encodes a glycoprotein precursor that is co-translationally processed into the surface glycoproteins Gn and Gc, as well as the nonstructural protein NSm. Gn and Gc assemble as heterodimers that form the viral envelope spikes and Gc is classified as a class II fusion protein [24]. Neutralizing antibody responses are directed primarily against epitopes within the surface-exposed head domain of Gc [27]. NSm is the least well-characterized of these proteins, and its function appears to be highly dependent upon the infected host with proposed roles ranging from assembly and morphogenesis to transmission and dissemination [28,29,30]. Together, proteins encoded on the S and M segments mediate viral entry, membrane fusion, assembly, intracellular trafficking and innate immune evasion, and therefore directly interface with host cell machinery. As such, they are likely primary determinants of viral fitness across both mosquito and mammalian hosts.

To investigate how viral genetics influence replication in mosquito and mammalian systems, we compared two distinct strains of JCV. JCV/61 is the prototypic 1961 isolate from Jamestown, Colorado, whereas JCV/03 is a more contemporary strain isolated in 2003 from pooled mosquitoes in Connecticut [31]. Initial characterization by Bennett and colleagues reported that the strains share 91%, 83%, and 85% nucleotide identity across the small, medium, and large segments, respectively, with amino acid identity ranging from 91–98% depending on the protein. Despite this high degree of genetic similarity, JCV/61 and JCV/03 have been reported to exhibit distinct phenotypes in weanling Swiss Webster mice [32]. This divergence suggests that relatively limited genetic differences underlie measurable phenotypic variation, making this strain pair a useful resource for identifying viral determinants of host-specific replication.

To identify JCV genetic determinants of host adaptation, we compared these two genetically similar JCV isolates in mammalian and insect cell lines. From this comparison, we identified a striking growth phenotype in mosquito-derived *Aedes albopictus* C6/36 cells that was unique to JCV/03. Furthermore, we found that a single passage in C6/36 cells conferred a heritable enhancement of JCV/03 replication in mosquito cells. We next sequenced mosquito cell-adapted JCV/03 and identified a small number of substitutions with the potential to enhance replication. Finally, we developed a plasmid-based system of reverse genetics that enabled precise manipulation of the JCV/03 genome and targeted investigation of the candidate mutations. Together, this work reveals specific mutations in the JCV/03 genome that modulate replication efficiency and multiple genetic pathways by which JCV can adapt to mosquito cells.

## 2. Materials and Methods

### 2.1. Cells and Viruses

Vero E6 cells (ATCC CRL-1586; ATCC, Manassas, VA, USA) and BSR-T7/5 cells were maintained in Dulbecco’s modified Eagle medium (DMEM) (Corning, Corning, NY, USA) supplemented with 10% cosmic calf serum (CCS) (HyClone, Cytiva, Marlborough, MA, USA) at 37 °C with 5% CO_2_. BSR-T7/5 cells were supplied by Carolina Lopez (Washington University School of Medicine, St. Louis, MO, USA) and maintained under selection with 2 mg/mL Geneticin (Invitrogen, Eugene, OR, USA), added every two or three passages. C6/36 cells (*Aedes albopictus*) were acquired from Sara Cherry (University of Pennsylvania) (ATCC CRL-1660) and cultured in Leibovitz’s L-15 medium (Gibco, Waltham, MA, USA) supplemented with 10% fetal bovine serum (FBS) (Corning), 1% penicillin-streptomycin (Gibco), and 1% GlutaMAX (Gibco) and incubated at 28 °C with 5% CO_2_. Primary rat cortical neurons were isolated from embryonic day 18 (E18) Sprague Dawley IGS rat pups (Charles River Laboratories, Wilmington, MA, USA) by the Penn Medicine Translational Neuroscience Core as previously described [33]. Neurons were maintained in Neurobasal medium (Gibco) supplemented with 2% B-27 (Invitrogen), 33 mM glucose, 40 mM HEPES (Gibco), and 1% penicillin-streptomycin and cultured in humidified chambers at 37 °C. Cultures were maintained for 7 days prior to use, with 40% of the medium replaced on day 4. All neuronal medium was conditioned at 37 °C with 5% CO_2_ prior to use.

Jamestown Canyon virus (JCV) strain 61V-2235 (NR-536) was obtained from the NIH Biodefense and Emerging Infections Research Resources Repository, and JCV/03/CT was provided by Philip Armstrong (The Connecticut Agricultural Experiment Station). Clonal populations of both viral strains were generated by three rounds of terminal dilution as describe by Bennett et al. [32]. Four biological clones of each virus were generated and sequenced as described below. Working virus stocks were propagated in Vero E6 cells and titrated by quantitative PCR (qPCR) or plaque assay on Vero E6 cells, as described below.

### 2.2. Sequencing of Purified Viral Clones

Four independently isolated biological clones of JCV/61 and JCV/03 were sequenced using a combination of cloning and PCR. Briefly, viral RNA was isolated from supernatant collected from infected cells using the RNeasy Mini Kit (QIAGEN, Cat #74104, Germantown, MD, USA) and Buffer RLT Plus (QIAGEN, Cat #1053393). cDNA was generated using the SuperScript™ III First-Strand Synthesis System (Invitrogen) and segment-specific primers. The same segment-specific primers used to synthesize the cDNA were also employed to generate the PCR product. A complete list of these primers is documented in Supplemental Table S1. All primers used in this study were synthesized by Integrated DNA Technologies (IDT) (Coralville, IA, USA).

To sequence the small segment, cDNA was amplified by PCR, then cloned into the pCRII-Blunt-TOPO plasmid using the Zero Blunt™ TOPO™ PCR Cloning Kit (Invitrogen, Cat #450245) according to the manufacturer’s protocol. To sequence the medium and large segments, cDNA was amplified by PCR and the linear amplicons were directly submitted for sequencing. Plasmids and linear amplicons were sequenced by Plasmidsaurus (Louisville, KY, USA) using Oxford Nanopore sequencing.

The generated consensus sequences were aligned to the existing reference genome using Geneious Prime (Version 2026.0.2) (Biomatters Ltd., Auckland, New Zealand). For each strain, the clone with the fewest mismatches to the reference sequence was selected as our prototype strain of JCV/61 (Small: U12796.1, Medium: U88058.1, Large: NC_043559.1) and JCV/03 (Small: HM007353.1, Medium: HM007354.1, Large: HM007355.1). The references for the small and medium segments of JCV/61 came directly from the data sheet supplied by BEI. The reference sequences for the large segment of JCV/61 and all three segments of JCV/03 were found in the literature [32]. Supplemental Table S2 summarizes differences between the reference and the chosen prototype strain. The consensus sequence of the prototype JCV/03 M segment generated in this study has been deposited in GenBank under accession number PZ903552.

### 2.3. Plaque Assays

Infectious units were determined by plaque assay on confluent Vero E6 cells in 6-well plates. Virus stocks were serially diluted 10-fold in serum-free DMEM, and 850 µL of each dilution was added per well. Virus was adsorbed for 1 h at 37 °C, after which the virus-containing medium was removed, cells were rinsed once with Dulbecco’s phosphate-buffered saline (DPBS) (Corning), and cells were overlaid with a 1:3 mixture of 2.5% Avicel RC-591 (DuPont, Wilmington, DE, USA) and complete DMEM. Plates were incubated at 37 °C for 5 days. 4% paraformaldehyde was added directly to each well to fix the cells. After 1 h, the Avicel-paraformaldehyde overlay was removed and cells were stained with 0.1% crystal violet (Sigma-Aldrich, Burlington, MA, USA) in 20% methanol to visualize plaques. Plaques were manually counted, and counts from replicate wells were averaged to calculate viral titers, which are reported as plaque-forming units per milliliter (PFU/mL).

### 2.4. Multistep Growth Curves in Vero, C6/36, and Primary Rat Cortical Neurons

Viral growth kinetics were assessed using multistep growth curves in three cell types. C6/36, Vero, and primary rat cortical neurons were seeded in 6-well plates at 3.15 × 10^5^, 2.3 × 10^5^, and 9.6 × 10^5^ cells per well, respectively. Neurons were grown on plates coated with 0.25 mg/mL poly-L-lysine hydrobromide (Sigma-Aldrich). One day after plating, cells were infected at multiplicities of infection (MOIs) of 0.1 or 0.03 in 2 mL of complete, cell-type-specific medium. The virus-media inoculum was not removed, and titers were reported as fold-change relative to zero hour post-infection. At each time point, a small sample of supernatant was collected and replaced with fresh, cell-type-specific media. Viral titers were quantified by plaque assay on Vero cells.

### 2.5. Analysis of Replication in C6/36 Cells

To analyze JCV replication in C6/36 cells, infections were performed in 6-well plates with an initial plating density of 1.8 × 10^6^ cells per well unless otherwise specified. Cells were incubated overnight at 28 °C with 5% CO_2_ and then infected with a target inoculum of 2 × 10^6^ genome copies per well in 2 mL of complete medium. C6/36 cells were minimally attached and prone to lifting, and due to the importance of cell density to viral growth kinetics, virus inoculum was not removed following infection. After infection, plates were returned to the incubator for the duration of the time course. At 0 hpi, an aliquot of the diluted inoculum was retained, and its genome-copy concentration was quantified by qPCR. The measured concentration was used to calculate the actual input genome copies per well, which served as the baseline for calculating fold-change rather than the intended inoculum of 2 × 10^6^ genome copies per well. The measured input values for each experiment are provided in Supplemental Table S3.

At each indicated time point, 75 µL of culture supernatant was collected and clarified by centrifugation at 1200× *g* for 5 min. An equal volume of fresh medium was added back to each well. Following clarification, 50 µL of supernatant was diluted into DPBS to a final volume of 140 µL and stored at −80 °C until RNA extraction.

For matched extracellular and intracellular time course experiments, the entire volume of cell supernatant was removed at each time point and clarified by centrifugation at 1200× *g* for 5 min. Following supernatant removal, cells were lysed directly in the culture wells by addition of 1 mL of TRIzol™ Reagent (Invitrogen). Clarified supernatant and cell lysates were stored at −80 °C until processing.

For variable-density growth curve experiments, cells were plated at high, medium, or low densities and infected with an equal number of plaque-forming units, resulting in MOIs of 0.03, 0.01, and 0.003, respectively. The supernatant was sampled over time as described above.

### 2.6. Analysis of Replication in Vero Cells

To analyze replication in Vero E6 cells, cells were plated at a density of 2 × 10^5^ cells per well in complete DMEM and incubated overnight at 37 °C with 5% CO_2_. Cells were infected with a target inoculum of 2 × 10^6^ genome copies per well in 2 mL of serum-free DMEM and incubated at 37 °C for 1 h to allow virus entry. The virus inoculum was then removed, cells were gently rinsed once with DPBS, and 2 mL of complete medium was added to each well. An aliquot of culture supernatant was collected immediately after medium replacement as the 0-hpi sample. Genome copies in this sample were quantified by qPCR and used as the baseline for calculating fold change. The measured 0-hpi values are provided in Supplemental Table S3. Plates were then returned to the incubator for the duration of the time course. In the serial passage experiments, the inoculum was not removed. At each indicated time point, 75 µL of culture supernatant was collected, clarified by centrifugation at 1200× *g* for 5 min, and replaced with an equal volume of fresh complete medium. Clarified supernatants were stored at −80 °C until RNA extraction.

### 2.7. RNA Extraction and cDNA Synthesis

Viral RNA was extracted from clarified supernatants using the QIAamp Viral RNA Mini Kit (Qiagen, Cat #52906) according to the manufacturer’s instructions and eluted in 30 µL of supplied AVE buffer. Total cellular RNA was isolated from TRIzol™ Reagent (Invitrogen) lysates and further purified using the Zymo RNA Clean & Concentrator Kit (Zymo Research, Cat #R1018, Irvine, CA, USA), with on-column DNase I treatment (Zymo Research, Cat #E1010). Purified cellular RNA was quantified using the Qubit RNA High Sensitivity Assay Kit (Invitrogen) on a Qubit 4 Fluorometer (Invitrogen).

For cDNA synthesis, either 8 µL of viral RNA from supernatants or equal amounts of total cellular RNA were reverse transcribed using the SuperScript™ III First-Strand Synthesis System (Invitrogen) with random primers (Invitrogen, Cat #4819011), following the manufacturer’s instructions.

### 2.8. Construction of JCV/03-pN Plasmid for qPCR Standard Curve

The JCV-pN expression plasmid used to generate the qPCR standard curve for absolute genome copy number quantification contains the JCV/03 nucleocapsid gene cloned into the pIRES-polyA backbone constructed by the authors (P. Bates, University of Pennsylvania). The pIRES-polyA plasmid backbone and JCV/03 nucleocapsid open reading frame were amplified using Q5 High-Fidelity 2X Hot Start Master Mix (New England Biolabs, Cat #M0494S, Ipswich, MA, USA) and the primers listed in Supplemental Table S4. Primer overhangs were designed to generate regions of homology required for HiFi DNA Assembly (NEB, Cat #M5520AA). The JCV/03 nucleocapsid PCR template was the gBlock™ (IDT, Coralville, IA, USA) used to construct the JCV/03-pS launch plasmid. The linearized backbone and PCR-amplified insert were assembled using NEBuilder HiFi DNA Assembly (NEB) according to the manufacturer’s instructions.

### 2.9. qPCR

Viral genome copy number in supernatant and cellular RNA was quantified by qPCR using PowerUp™ SYBR™ Green Master Mix (Applied Biosystems, Waltham, MA, USA) and JCV-specific primers. Primers targeted the small genome segment and were designed to bind identical regions in both strains to ensure comparable amplification efficiency. Supplemental Table S5 lists the qPCR primers used in this study.

Absolute genome copy number was quantified using a six-point standard curve generated from 10-fold serial dilutions of the JCV/03-pN expression plasmid. Plasmid concentration was converted to copy number using a molecular weight-based equation [34]. qPCR was performed on a QuantStudio 6 Flex Real-Time PCR System (Applied Biosystems) using the following cycling conditions: initial incubation at 50 °C for 2 min and 95 °C for 10 min, followed by 40 cycles of 95 °C for 15 s, 52 °C for 30 s, and 72 °C for 30 s, with fluorescence acquisition during the 52 °C and 72 °C steps. Standard curves generated by dilution of JCV/03-pN had a calculated R^2^ between 0.9808 and 0.9994. The qPCR efficiency ranged from 81% (R^2^ = 0.996) to 98% (R^2^ = 0.9808).

For samples derived from cell lysates, values were normalized to actin, using a ΔCt-based correction (Ct_actin–Ct_ref, where Ct_ref is the time-matched mock), yielding housekeeping-adjusted genome copies per µg RNA. For samples derived from culture supernatants, normalization to a housekeeping gene was not possible; therefore, viral genome copies per milliliter were calculated based on the volume of supernatant collected and the volume of cDNA input, assuming uniform recovery and reaction efficiency across samples and processing steps.

### 2.10. RNA Sequencing and Alignment

Viral RNA was extracted from clarified cell culture supernatants using TRIzol™ LS Reagent (Invitrogen) and purified using the Zymo RNA Clean & Concentrator kit (Zymo Research). Purified RNA was submitted to the University of Pennsylvania School of Veterinary Medicine Center for Host–Microbial Interactions for library preparation and sequencing. cDNA libraries were prepared using the Illumina^®^ Stranded Total RNA Prep Ligation with Ribo-Zero Plus kit (Illumina, Cat #20040529, San Diego, CA, USA) and sequenced on an Illumina NextSeq 2000 platform using a P2 100-cycle kit (Illumina, Cat #20046811).

Raw sequencing reads were aligned to the published Jamestown Canyon virus reference genome (Small: HM007353.1, Medium: HM007354.1, Large: HM007355.1) using breseq (v0.36.0) in polymorphism mode, which assumes a mixed population of viral variants. Read mapping was performed using Bowtie2 (v2.4.1), and mutations were identified relative to the reference genome using a minimum variant frequency cutoff of 5%. The breseq gdtools compare function was used to generate comparative mutation frequency reports across samples [35].

### 2.11. Construction of JCV/03 Launch Plasmids

Launch plasmids encoding the JCV small, medium, and large genome segments were constructed using a modular DNA assembly approach. A T7-driven plasmid backbone containing the T7 optimal promoter, super-cutter hepatitis delta virus (HDV) ribozyme, and T7 terminator was used to clone the S, M and L segment inserts. Viral genome sequences corresponding to JCV/03 (Small: HM007353.1, Medium: HM007354.1, Large: HM007355.1) were synthesized as double-stranded DNA fragments (gBlocks™, IDT) containing the antigenomic sequence of each genome segment with the addition of the hammerhead ribozyme sequence positioned at the 5′ end. The complete S segment was synthesized as a single fragment, the M segment as two fragments, and the L segment as four fragments. All fragments were designed with overlapping regions compatible with seamless assembly. Plasmids pS, pM, and pL were assembled by combining the linearized plasmid backbone with the corresponding PCR-amplified gBlocks™ using NEBuilder HiFi DNA Assembly (NEB) according to the manufacturer’s instructions. Final constructs were verified by Oxford Nanopore sequencing (Eurofins Genomics, Louisville, KY, USA).

### 2.12. Construction of M Segment Mutants

Individual point mutations within the pM plasmid were introduced by PCR-based mutagenesis using primers listed in Supplemental Table S6, followed by circularization using NEBuilder HiFi DNA Assembly (NEB). All PCR reactions described in this manuscript were performed using Q5 High-Fidelity 2X Hot Start Master Mix (NEB) according to the manufacturer’s instructions.

### 2.13. Rescue of Recombinant Virus

Rescue of recombinant virus was performed in BHK BSR-T7/5 cells using Lipofectamine L3000 (Invitrogen, Cat #L3000015). Cells were seeded at a density of 1.8 × 10^5^ cells/mL and transfected with equal amounts (0.5–2 µg each) of plasmids JCV/03-pS, -pM, and -pL. Transfections were carried out according to the manufacturer’s instructions, using 1 µL Lipofectamine 3000 and 1 µL P3000 per µg of total DNA. 3–5 days post-transfection, cells and supernatants were pooled with approximately 1 × 10^5^ Vero cells, plated with fresh growth medium in a 10 cm dish, and returned to the incubator. When roughly 70% cytopathic effect was observed, cultures were harvested, clarified, and aliquoted as passage 0 (P0) virus stocks. Passage 1 (P1) stocks were generated by amplification on Vero cells and experiments were performed with P1 viral stocks.

To verify successful rescue of recombinant viruses containing point mutations, viral RNA was extracted from P0 and P1 stocks and used for cDNA synthesis as described above. cDNA was used as a template for PCR targeting regions within the M segment containing the mutation of interest, using the primers in Supplemental Table S1. Linear amplicons were submitted to Eurofins (Eurofins Genomics, Louisville, KY, USA) for Oxford Nanopore sequencing.

### 2.14. In Silico Structural Modeling

The predicted three-dimensional structure of JCV/03 Gn was generated using AlphaFold 3 through the AlphaFold Server (alphafoldserver.com) (Google DeepMind and Isomorphic Labs, London, UK). The amino acid sequence of the JCV/03 M-segment polyprotein was obtained from GenBank (accession no. ADK36673.1). Gn was defined as residues 14–301 of the M-segment polyprotein based on the coding-region boundaries described by Bennett et al. [32]. Models were generated using the JCV/03 M-segment reference sequence with the polyprotein residue 193 specified as either serine or glycine. Each Gn sequence was submitted as a single protein chain using the default server settings. The top-ranked model displayed by the server was used for visualization. Models were manually rotated within the AlphaFold Server viewer to provide approximately comparable orientations, and images were captured directly from the viewer. No coordinate-based structural alignment or additional refinement was performed.

### 2.15. Statistical Analysis

Only growth curves with three replicate culture wells per condition were analyzed for statistical significance. Technical qPCR replicates were averaged to obtain one value for each culture well at each time point. For comparing genome copies in supernatant to intracellular copies, log10-transformed values were analyzed by ordinary two-way ANOVA with Šídák’s multiple-comparisons test to compare JCV/61 and JCV/03 at each time point. For all other analyzed growth curves, genome copies/mL or fold-change values were log10-transformed, and area under the curve (AUC) was calculated separately for each well across the full time course with a baseline of Y = 0. AUC values were compared using two-tailed paired *t*-tests, with wells matched by well number across conditions. Data are presented as the mean ± SD. Statistical significance is indicated as * *p* < 0.05, ** *p* < 0.01, *** *p* < 0.001, and **** *p* < 0.0001. Analyses were performed using GraphPad Prism 11.0.2 (92) (GraphPad Software, Boston, MA, USA).

## 3. Results

### 3.1. JCV/03 Displays a Mosquito Cell-Specific Replication Delay

**Figure 1:**
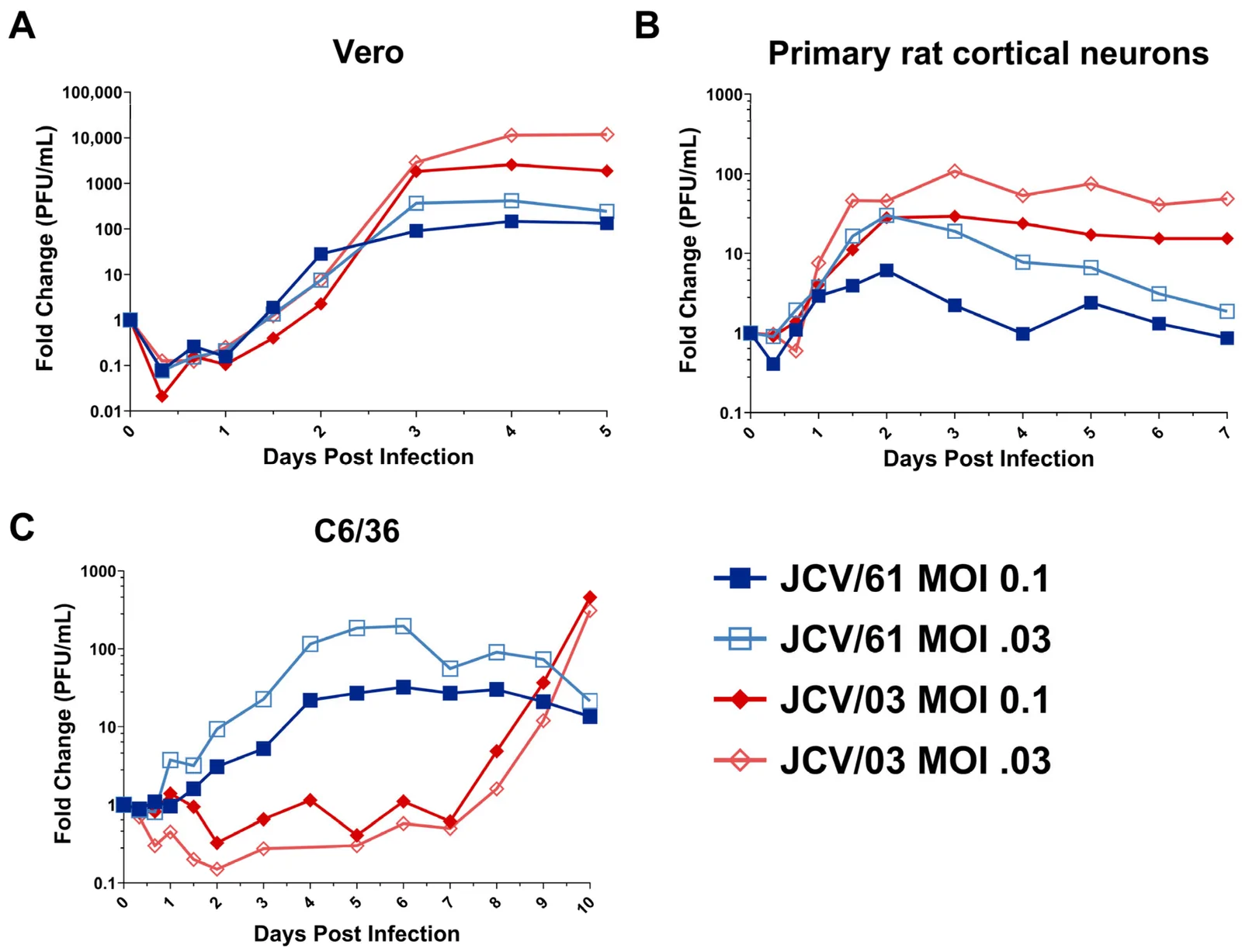
JCV/03 exhibits a distinct lag-burst growth phenotype in C6/36 cells. (**A**) Vero cells, (**B**) primary rat cortical neurons, and (**C**) C6/36 cells were infected with two strains of JCV at MOIs of 0.1 and 0.03. Supernatant was collected at the indicated time points and infectious virus was quantified by plaque assay on Vero cells. Titers are expressed as fold change in PFU/mL relative to input. One biological replicate was analyzed per MOI, with one sample collected at each time point.

JCV/03 and JCV/61 had previously been reported to exhibit different in vivo pathology phenotypes, with JCV/61 being more neuropathogenic. However, the strains were reported to behave similarly when analyzed in vitro [32]. To begin our evaluation of these viruses, we performed limited dilution cloning on Vero E6 cells to produce uniform viral stocks. One clone for each virus strain was then chosen for analysis of growth in vitro in three cell lines. African green monkey Vero E6 cells, mosquito-derived *Aedes albopictus* C6/36 cells, and primary rat cortical neurons were infected with JCV/61 or JCV/03 at multiplicity of infection (MOI) 0.1 and 0.03, and released virus was sampled at regular intervals for the duration of cell viability. Viral titer at each time point was quantified by plaque assay on Vero E6 cells. In Vero cells, JCV/03 and JCV/61 exhibited similar replication kinetics. The replication curve profiles were comparable at both MOIs, with JCV/03 ultimately reaching a higher peak titer than JCV/61 (Figure 1A). In primary rat cortical neurons, both viruses initially replicated with similar kinetics, reaching a peak titer at day 2. As seen with replication in Vero cells, JCV/03 titers were higher than those of JCV/61. Additionally, JCV/61 titers declined at day 3 due to neuronal cell death, while JCV/03 titers remained relatively constant for the duration of the time course through day 7, at which point the neuronal cells began losing viability (Figure 1B).

In contrast to what is observed in mammalian cells, replication in mosquito C6/36 cells revealed a striking strain-specific difference between JCV/61 and JCV/03 (Figure 1C). JCV/61 replicated steadily, reaching peak titers by approximately 4 days post-infection and subsequently plateauing. JCV/03, however, exhibited minimal accumulation of infectious virus for seven days post-infection, followed by an abrupt transition to rapid replication. During the final three days of the time course, JCV/03 titers increased nearly 1000-fold, ultimately surpassing JCV/61. This “lag-burst” pattern was identical between the two MOIs. Given this unusual behavior, we analyzed replication in mosquito cells for three additional purified clones of JCV/03 and the uncloned parental stock that was obtained from The Connecticut Agricultural Experiment Station. All four clones and the parental stock displayed a six- to eight-day delay in replication followed by a dramatic replication increase (Supplemental Figure S1). Thus, the unusual phenotype for JCV/03 replication in mosquito cells is an inherent feature and not restricted to a selected clone. Overall, these results establish that while JCV/03 and JCV/61 replicate similarly in mammalian cells, JCV/03 exhibits a distinct lag-burst phenotype exclusively in insect C6/36 cells.

### 3.2. The JCV/03 Lag-Burst Phenotype Reflects Delayed Viral RNA Replication

**Figure 2:**
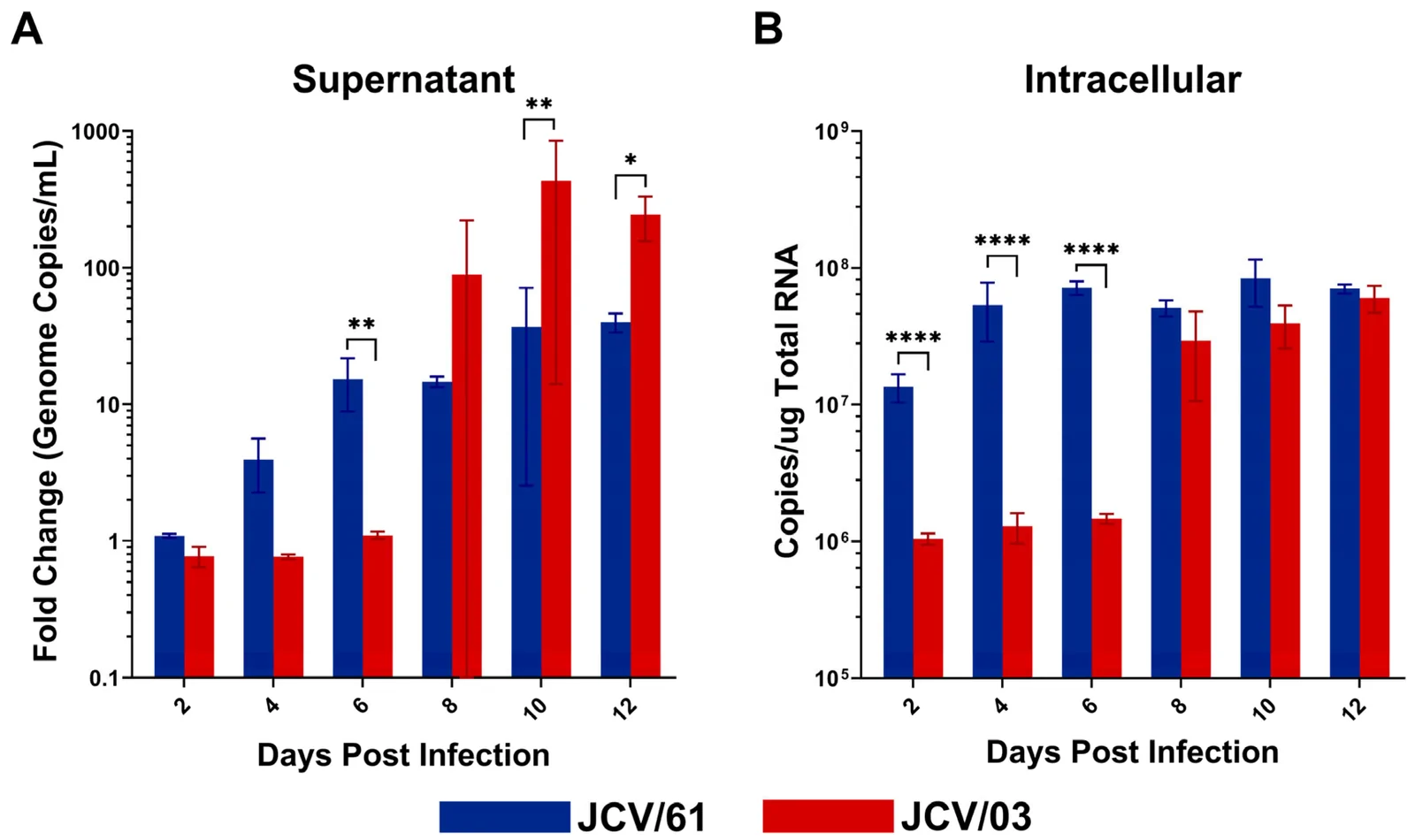
The JCV/03 lag-burst phenotype reflects delayed viral replication. C6/36 cells were infected with JCV/61 or JCV/03 using a target inoculum of 2 × 10^6^ genome copies per well. (**A**) Extracellular viral RNA was quantified from culture supernatants by qPCR and expressed as fold change in genome copies per mL relative to the corresponding input measured by qPCR at 0 hpi. Measured input values are provided in Supplemental Table S3. (**B**) Intracellular viral RNA was quantified from total cellular RNA by qPCR, normalized to C6/36 actin, and expressed as genome copies per µg total RNA. Data represent the mean of three replicate wells from one independent experiment. Error bars represent ± SD. Each culture sample was measured in technical triplicate by qPCR. Statistical significance between viruses at each time point was determined by ordinary two-way ANOVA with Sidak’s multiple comparisons test on log10-transformed values. * *p* < 0.05, ** *p* < 0.01, **** *p* < 0.0001.

Analysis of JCV/03 virus in the supernatant of infected insect cells revealed an unusual delay in the appearance of infectious virus. Therefore, we wanted to address whether JCV/03 was replicating intracellularly but failing to efficiently assemble or exit cells. C6/36 cells were infected with equal genome copies of JCV/61 or JCV/03, and at each time point the entire supernatant and corresponding cell lysates were collected for quantification of viral RNA. As observed previously for infectious virus, JCV/61 genomic RNA in the supernatant increased steadily over time, whereas the release of JCV/03 genomic RNA was delayed until day 8 when it rapidly increased (Figure 2A, compare blue vs. red bars). qPCR analysis of intracellular RNA revealed a pattern similar to that observed for released virus. JCV/61 genomic RNA reached peak levels early on and remained stable for the duration of the time course (Figure 2B, blue bars). As was seen for extracellular virus, JCV/03 showed minimal intracellular viral RNA accumulation during the first 6 days, followed by a rapid increase at day 8 to levels comparable to JCV/61, after which JCV/03 plateaued (Figure 2B, red bars). Therefore, the delayed kinetics of JCV/03 were evident both intracellularly and extracellularly, indicating that the lag phase reflects delayed viral replication rather than a lag in viral assembly or egress.

### 3.3. Plating Density Modulates Timing and Magnitude of JCV/03 Replication in C6/36 Cells

**Figure 3:**
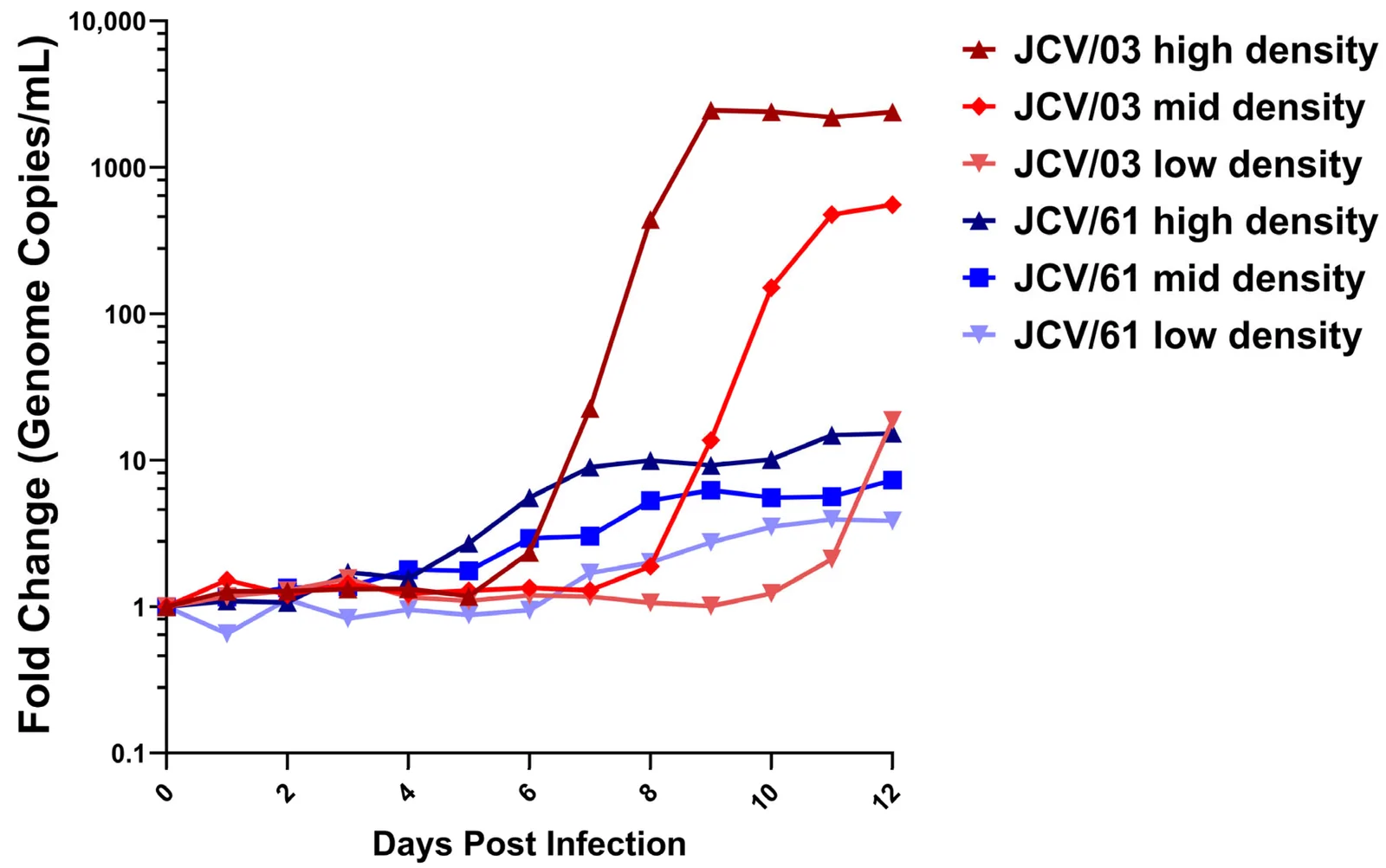
Cell density modulates JCV/03 replication kinetics in C6/36 cells. C6/36 cells were seeded in 6-well plates at low (2 × 10^5^ cells per well), medium (6 × 10^5^ cells per well), or high (1.8 × 10^6^ cells per well) density and infected with 6000 PFU of JCV/61 or JCV/03. The resulting MOIs were 0.03, 0.01, and 0.003 for low-, medium-, and high-density wells, respectively. A single culture well was used for each virus at each plating density, and one supernatant sample was collected from that well at each time point. An aliquot of each inoculum was retained at 0 hpi, and the actual input genome copies per well were quantified by qPCR (Supplemental Table S3). Supernatants were collected at the indicated time points, and viral RNA was quantified by qPCR to determine genome copies per well. Values are expressed as fold change relative to the qPCR-quantified 0-hpi input.

The abrupt transition of JCV/03 to rapid replication suggested that an extrinsic, time-dependent factor might govern viral growth, such as cell–cell proximity, abundance or accumulation of secreted factors that affect cellular physiology. We therefore hypothesized that increasing the initial cell density would accelerate the transition to rapid viral replication. To address whether cellular density affected JCV/03 replication, C6/36 cells were plated at low, medium, or high density and infected with a fixed quantity of infectious JCV/61 or JCV/03. JCV/61 replication was consistent across conditions and growth curves segregated according to initial plating density, with higher-density wells yielding higher overall genome accumulation but no qualitative change in replication kinetics (Figure 3, blue curves). By contrast, JCV/03 replication was strongly influenced by plating density. Increasing cell density progressively shortened the lag phase, however after the lag, the slope of the curves appeared similar. The plating density also appeared to alter the magnitude of replication. The highest-density condition exhibited the earliest transition to exponential growth at day 6 and reached the highest peak viral titer. This peak titer was approximately 100-fold higher than that of JCV/61 plated at the same cell density (Figure 3, dark red vs. dark blue curves). These data demonstrate that cell density is directly correlated with both the timing and magnitude of the JCV/03 lag-burst phenotype, although the mechanistic basis of this relationship remains undefined.

**Figure 4:**
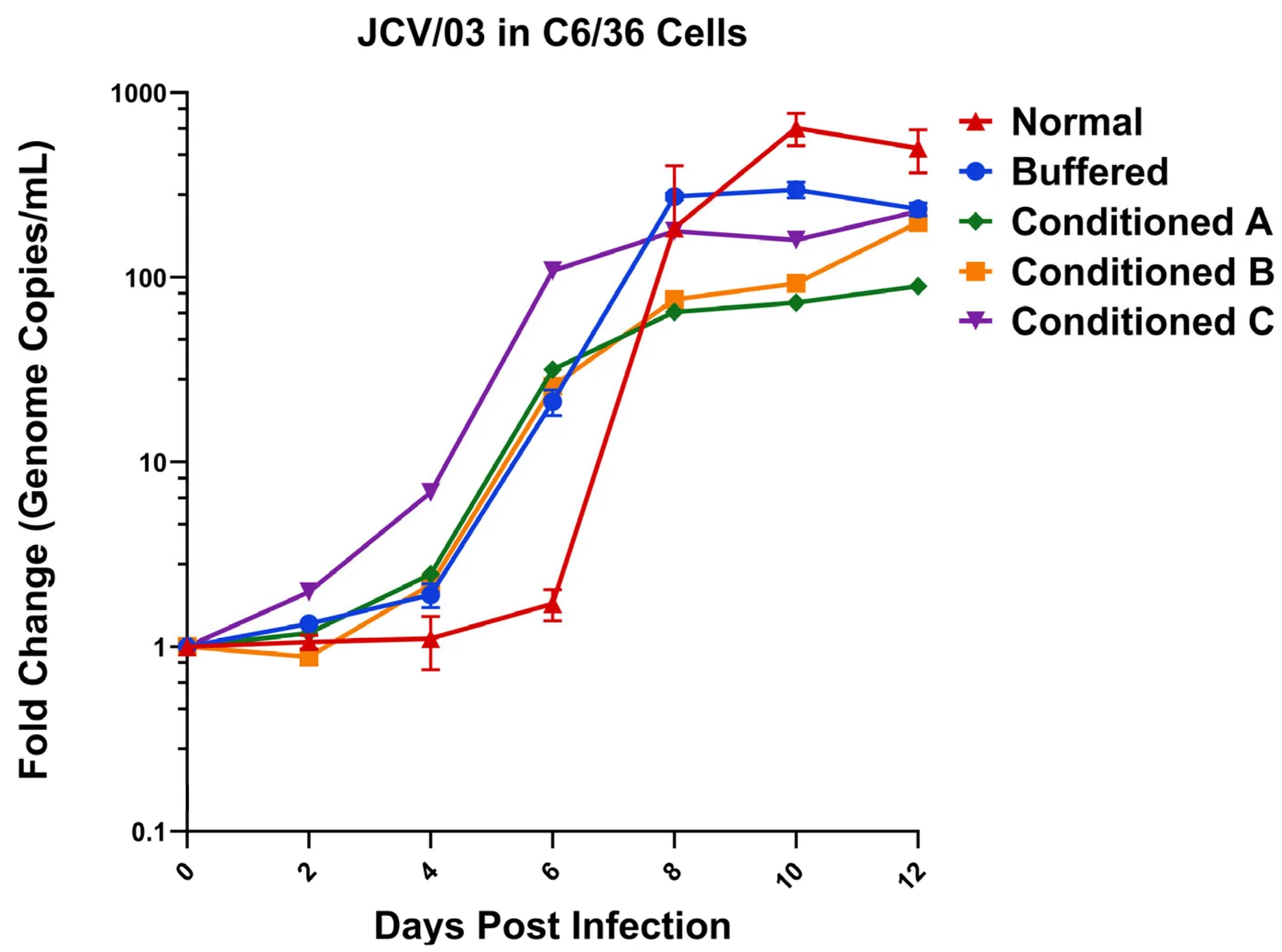
Altering media conditions modulates JCV/03 replication kinetics in C6/36 cells. C6/36 cells were infected with 2 × 10^6^ genome copies of JCV/03 in standard growth medium, growth medium buffered with 25 mM HEPES, or conditioned medium collected from high-density mock-infected C6/36 cultures. Three batches of conditioned media were tested. Viral RNA in supernatant was quantified by qPCR and values are expressed as fold change relative to input. One biological replicate was analyzed for each batch of conditioned medium, and two biological replicates were analyzed for the standard and buffered medium conditions. Error bars represent ± SD.

To further investigate extracellular factors governing the lag-burst replication phenotype, infections were performed in C6/36 cells cultured in buffered or conditioned media. Buffered media consisted of standard C6/36 growth media supplemented with HEPES to a final concentration of 25 mM. The conditioned media was prepared by plating C6/36 cells at high density and collecting the media 12 days after mock infection. Three independent batches of conditioned media were tested. In standard media, JCV/03 exhibited minimal replication through day 6 post-infection, rapidly reached peak titers by approximately day 8, and then remained stable through day 12 (Figure 4, red line). In buffered media, the lag phase decreased modestly, with titers beginning to increase by day 4 post-infection; however, the virus still reached peak titers on day 8 and to the same magnitude as with standard media (Figure 4, blue line). In conditioned media batches A and B, the replication lag was similarly shortened, with titers increasing by day 4 and plateauing by day 8. Due to cytotoxicity induced by the conditioned media, peak titers were approximately ten-fold lower than those observed in standard or buffered media (Figure 4, green and orange lines). In contrast, conditioned media batch C displayed no lag in replication, but the pace was gradual rather than exponential, with titers steadily increasing through day 6 before plateauing (Figure 4, purple line). Together, these findings indicate that while extracellular conditions can influence the timing and magnitude of JCV/03 replication in C6/36 cells, they are insufficient to eliminate the lag-burst pattern, suggesting that this replication phenotype is primarily driven by intrinsic viral determinants.

### 3.4. A Single Passage in C6/36 Cells Confers Heritable Enhancement of JCV/03 Replication

**Figure 5:**
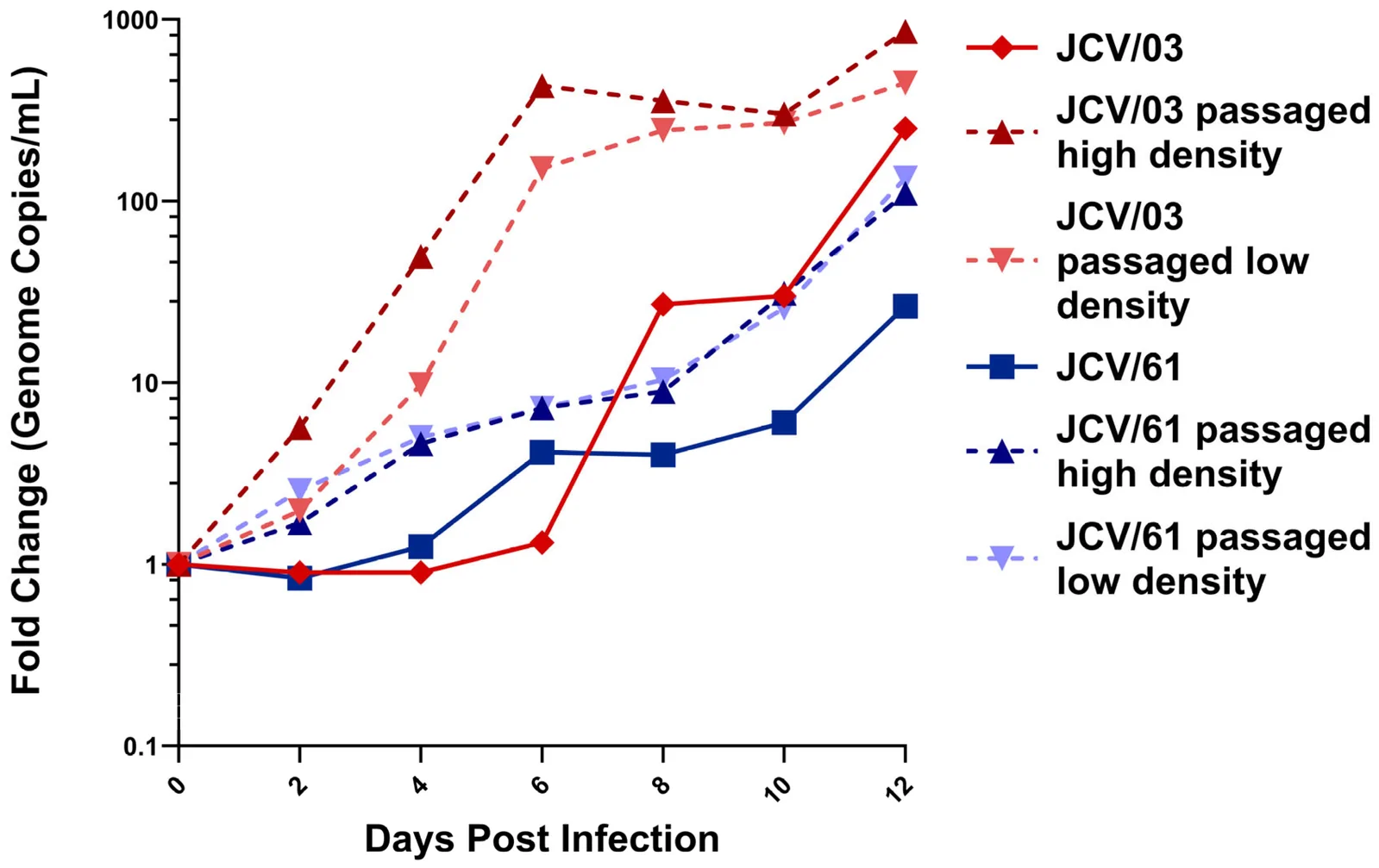
A single passage in C6/36 cells confers heritable enhancement of JCV/03 replication. C6/36 cells were infected with parental JCV/03 or JCV/61, or with parental virus passaged once in C6/36 cells at low or high density, using a target inoculum of 2 × 10^6^ genome copies. A single culture well was used for each virus and one supernatant sample was collected from that well at each time point. Supernatants were collected at the indicated time points, and viral RNA was quantified by qPCR to determine genome copies per mL. Values are expressed as fold change in genome copies per mL relative to the corresponding input measured by qPCR at 0 hpi. Measured input values are provided in Supplemental Table S3.

We next asked whether the abrupt increase in JCV/03 replication in C6/36 cells reflected a heritable change and whether this adaptation depended on the initial cellular plating density. To address this, parental stocks of JCV/03 and JCV/61 were used to infect C6/36 cells plated at high and low densities, and virus was collected from these cultures on day 12. Equal genome copies of these mosquito-derived (passaged) virus stocks were then used to infect fresh C6/36 cells plated at high density. For comparison, C6/36 cells were also infected with equal genome copies of the parental JCV/61 or JCV/03 stocks that were derived from Vero cells. Mosquito cell-derived JCV/61 virus exhibited a modest enhancement of replication when used to infect fresh C6/36 cells (Figure 5, dashed blue lines). Compared to Vero-derived virus, replication of JCV/61 from mosquito cells was slightly accelerated and peak titers at day 12 were approximately 5-fold higher (Figure 5, compare solid and dashed blue lines), but overall the replication pattern was unchanged.

In contrast, JCV/03 collected from both high- and low-density C6/36 cultures replicated immediately and exponentially when infecting fresh C6/36 cells, with no detectable delay (Figure 5, dashed red lines). Peak titers were reached by day 6 and leveled off. As seen before, Vero-derived JCV/03 displayed a distinct replication lag, transitioned to rapid replication on day 6, and then plateaued through the end of the 12-day experiment (Figure 5, solid red line). Collectively, these data demonstrate that JCV/03 acquires heritable, enhanced mosquito cell replication after a single passage in C6/36 cells, regardless of the initial plating density.

### 3.5. Serial Passage Reveals Distinct Evolutionary Trajectories of JCV/03 Adaptation

**Figure 6:**
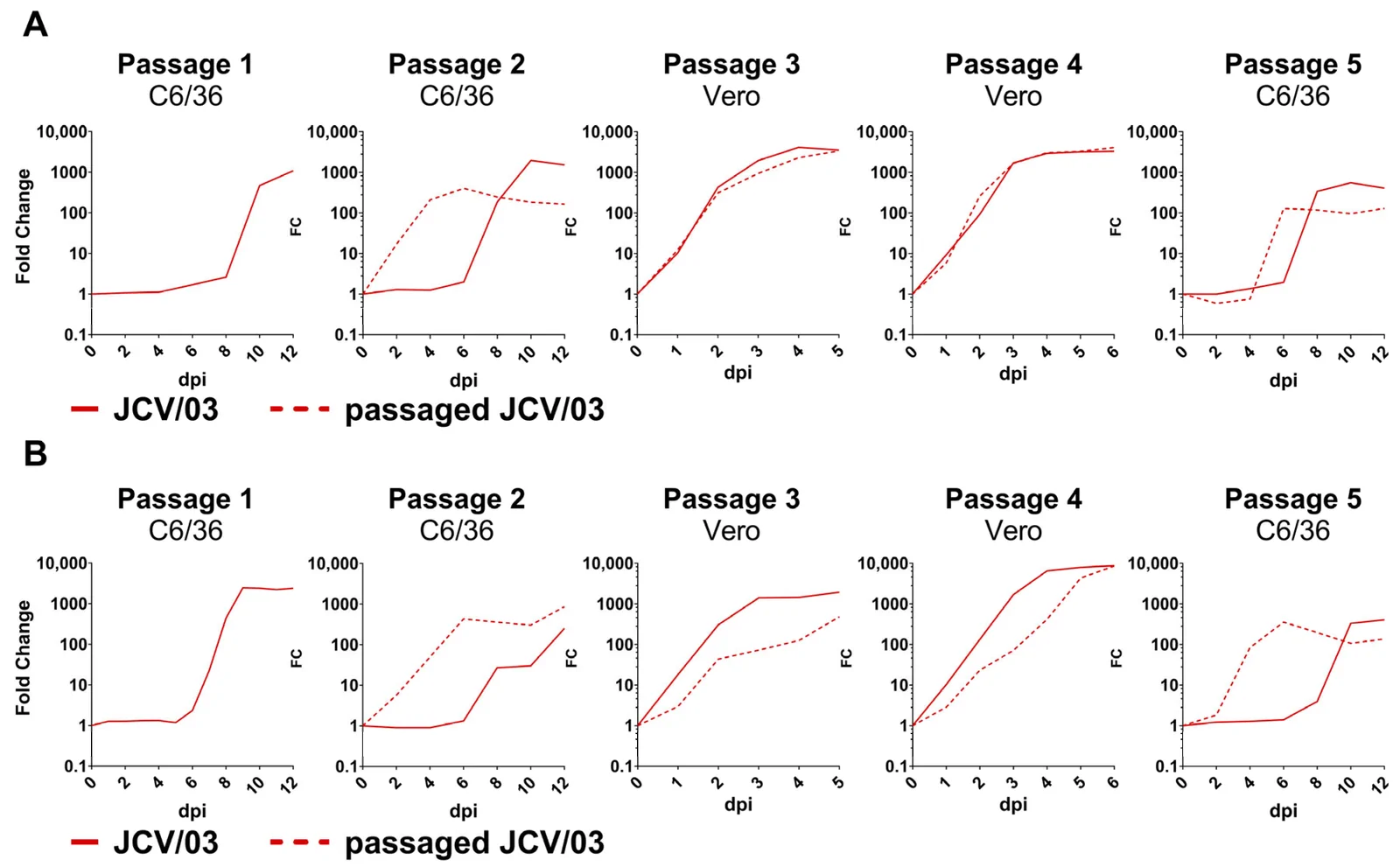
Serial passage reveals consistent adaptation in insect cells but divergent outcomes after mammalian passage. Two independent five-passage series were performed. In each series, C6/36 or Vero cells were infected with either parental JCV/03 (solid line) or passaged JCV/03 (dashed line) using a target inoculum of 2 × 10^6^ genome copies. JCV/03 was passaged twice in C6/36 cells (Passages 1 and 2), followed by two passages in Vero cells (Passages 3 and 4), and subsequently returned to C6/36 cells (Passage 5). To generate growth curves for Series I (**A**) and Series II (**B**), supernatants were collected every other day during C6/36 time courses and daily during Vero time courses. A single culture well was used for each virus in each passage, with one supernatant sample collected at each time point. Viral RNA was quantified by qPCR to determine genome copies per mL. Values are expressed as fold change (FC) in genome copies per mL relative to the corresponding input measured by qPCR at 0 hpi. Measured input values are provided in Supplemental Table S3.

Given that a single passage of JCV/03 in C6/36 cells conferred a heritable replication advantage in C6/36 cells, we next asked whether this adaptation affected viral replication in mammalian cells and whether the advantage would persist or revert following passage in mammalian cells. To address these questions, we performed two independent serial passage experiments and analyzed the viral phenotype and genotype after each passage. In each series, JCV/03 was passaged twice in high-density C6/36 cells, followed by two passages in Vero cells, and a fifth passage back into C6/36 cells (Figure 6A,B). The parental stock of Vero-derived JCV/03 was employed as the control for each set of infections (Figure 6A,B, solid red lines). During the first set of serial passages (Series I), Vero-derived JCV/03 displayed the characteristic replication lag followed by a rapid increase at day 8 (Figure 6A, first panel). As seen previously, mosquito cell-derived JCV/03 taken from passage 1 showed no replication lag when infecting fresh C6/36 cells (Figure 6A, second panel dashed line). Replication of JCV/03 in Vero cells during passages three and four was identical to that of parental, Vero-derived virus (Figure 6A, dashed lines third and fourth panels). However, when the virus from Vero cells in panel 4 was returned to C6/36 cells, replication had reverted to the original lag-burst phenotype observed for parental JCV/03 (Figure 6A, panel five).

The second serial passage experiment (Series II) also showed viral adaptation to mosquito cells during passage 1, resulting in the absence of a lag phase when this stock was used to infect fresh C6/36 cells for passage 2 (Figure 6B, dashed line second panel). However, in contrast to the results from Series I, adaptation to C6/36 cells was accompanied by subsequent impaired replication in Vero cells, where mosquito cell-passaged virus replicated more slowly than parental JCV/03 (Figure 6B, dashed lines third and fourth panels). Additionally, unlike what was observed in Series I, the Series II virus did not revert after two passages in Vero cells. When returned to C6/36 cells in passage five, replication was rapid and the lag phase was significantly abbreviated (Figure 6B, panel 5 dashed line). Overall, these results confirm that heritable changes occur in JCV/03 upon growth in mosquito cells and these changes can either remain stable (Series II) or revert (Series I) upon growth in mammalian cells.

To determine whether initial plating density influenced the outcome of serial passage, we repeated the five-passage experiment using virus initially adapted in low-density C6/36 cultures (Supplemental Figure S2). Passage 1 was performed with an initial plating density approximately six-fold lower than the high-density conditions used in Figure 6, while passages 2–5 were performed as described above. Similarly to virus adapted using high-density mosquito cell cultures, low-density-derived JCV/03 displayed immediate replication upon reinfection into C6/36 cells, with no apparent lag phase (Supplemental Figure S2, panel 2). Although replication in passage 3 was comparable to that of parental Vero-derived virus, growth was noticeably reduced by passage 4, resembling the phenotype observed for the high-density virus in Series I (Supplemental Figure S2, panel 3 and panel 4). When returned to C6/36 cells for passage 5, the virus retained an abbreviated lag phase and accelerated replication kinetics, indicating that adaptation to insect cells persisted, at least partially, despite two passages in mammalian cells. This shortened lag is similar to that observed in the Series II passages. Together, these results demonstrate that the initial plating density does not substantially alter the long-term outcome of serial passage.

### 3.6. A Plasmid-Based Reverse Genetics System Reveals That S193G Is Required for the JCV/03 Phenotype

To reveal changes responsible for adaptation to mosquito cells, JCV/03 viral RNA from the supernatant at the end of each passage (Figure 6, day 12 on insect cells and day 5 or 6 on Vero cells) was extracted and sequenced. Reads were aligned to the published JCV/03 reference genome (Small: HM007353.1, Medium: HM007354.1, Large: HM007355.1). During this analysis we identified a variation in the M segment region encoding Gn (S193G) that was fixed in our parental stock of JCV/03 and present in all four JCV/03 clones purified by limiting dilution but absent from the published reference genome [32]. Upon growth in either Vero or mosquito cells, the S193G variant remained stable in both Series I and Series II. Because this change was present at 100% frequency in our parental JCV/03 stock and maintained at 100% throughout the serial passage experiments, we sought to determine if S193G contributed to the delayed replication phenotype observed in mosquito cells.

To investigate the importance of the changes identified in JCV/03, we employed a plasmid-based reverse genetics system to generate recombinant viruses with precisely defined genomes. In this system, T7-driven plasmids encoding each genome segment were co-transfected into BHK BSR-T7/5 cells, which constitutively express T7 RNA polymerase [36]. The launch plasmids contained the full-length antigenomic cDNA of a genome segment, including the UTRs, positioned between an optimized T7 promoter and T7 terminator [37]. To ensure precise termini of the viral RNA transcripts, plasmids were constructed with self-cleaving ribozymes flanking the genome segment. A hammerhead ribozyme was positioned upstream of the viral genome segment to generate the correct 5′ end, while a super-cutter hepatitis delta virus (HDV) ribozyme was placed downstream to produce the correct 3′ end [38,39]. Following transfection, the T7 RNA polymerase expressed in BSR-T7/5 cells transcribed antigenomic cDNA from each plasmid, initiating translation of viral proteins and production of infectious virus. After launch, stocks of the recombinant viruses were produced by one round of replication in Vero cells.

**Figure 7:**
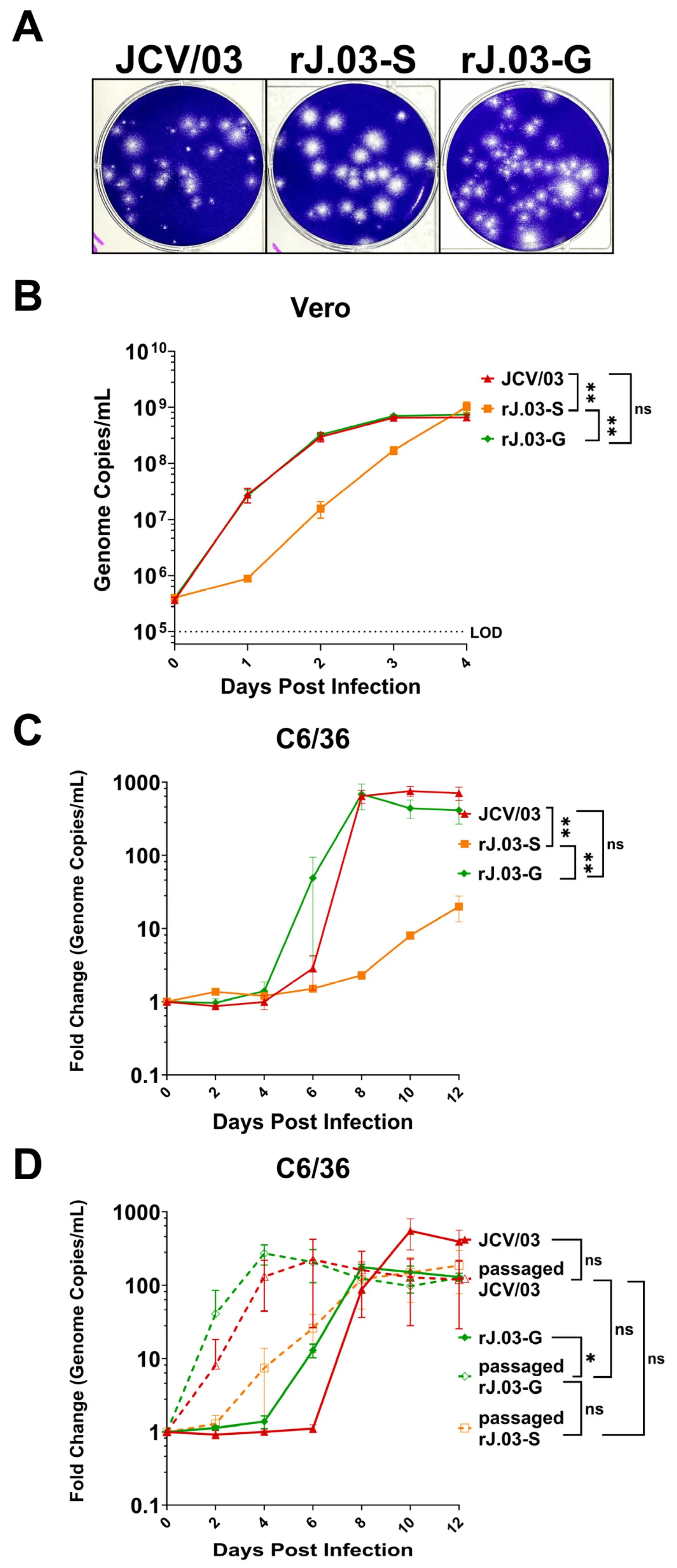
S193G is required for the parental JCV/03 lag-burst phenotype. (**A**) Representative plaque morphology of parental JCV/03, recombinant JCV/03 encoding serine at residue 193 (rJ.03-S), and recombinant JCV/03 encoding glycine at residue 193 (rJ.03-G) after 5 days on Vero E6 cells. Multistep growth curves of parental JCV/03, rJ.03-S, and rJ.03-G in (**B**) Vero E6 and (**C**) C6/36 cells. (**D**) Growth of unpassaged parental JCV/03 and rJ.03-G and passaged JCV/03, rJ.03-S, and rJ.03-G in C6/36 cells. Cells were infected with a target inoculum of 2 × 10^6^ genome copies, and viral RNA in supernatants collected at the indicated time points was quantified by qPCR. Titers in (**C**,**D**) are expressed as fold change in genome copies/mL relative to the corresponding input measured at 0 hpi. Measured inputs are provided in Supplemental Table S3. Growth curves show the mean ± SD of three replicate culture wells from one experiment. Each sample was quantified in technical triplicate, and technical replicates were averaged before analysis. For statistical analysis, genome copies/mL in (**B**) and fold-change values in (**C**,**D**) were log10-transformed. Area under the curve was calculated separately for each well across the full time course with a baseline of Y = 0. Comparisons were made using two-tailed paired *t*-tests, with wells matched by well number across conditions. *p* values are indicated as * *p* < 0.05, ** *p* < 0.01. The dotted line in (**B**) indicates the limit of detection.

Recombinant JCV/03 viruses encoding serine at position 193 (rJ.03-S), as specified by the published reference sequence, or glycine at position 193 (rJ.03-G), as found in our parental stock, were launched and characterized. We compared our stock JCV/03, rJ.03-S, and rJ.03-G by plaque assay and multistep growth curves in Vero and C6/36 cells. All three viruses produced mixed-size plaques on Vero cells (Figure 7A). Growth curves in both Vero and C6/36 cells revealed a significant difference between the two recombinant viruses. As might be expected, the rJ.03-G virus, which is identical in sequence to the parental JCV/03 stock, replicated similarly to the parental virus in both mammalian (Figure 7B) and insect cells (Figure 7C). By contrast, rJ.03-S replicated more slowly in both insect and mammalian cells (orange curves, Figure 7B,C). Notably, in C6/36 cells rJ.03-S did not exhibit the characteristic lag-burst phenotype observed for parental JCV/03, while rJ.03-G displayed the parental phenotype (Figure 7C). Sequence comparison showed that glycine is highly conserved at this position across orthobunyaviruses, including JCV/61 and all other available JCV isolates, supporting the interpretation that S193 represents an unusual and potentially poorly tolerated substitution. Moreover, the difference between the deposited JCV/03 sequence and our virus stocks can be explained by a single nucleotide change, raising the possibility that S193 reflects a sequencing error in the published reference. Overall, these results demonstrate that a serine at position 193 is detrimental for replication in both mammalian and insect cells. Moreover, only JCV/03 with glycine in this position displays the lag-burst in mosquito cells.

To determine whether S193G was also required for the heritable adaptation observed in C6/36 cells or only necessary for the initial lag-burst phenotype, we compared the growth of C6/36-derived rJ.03-S, rJ.03-G, and JCV/03 with Vero-derived JCV/03 and rJ.03-G (Figure 7D, compare dashed and solid lines). As seen previously, passage of parental JCV/03 in C6/36 mosquito cells abolishes the replication lag seen with virus derived from mammalian cells (Figure 7D, compare solid and dashed red lines). Similarly, passage of rJ.03-G in C6/36 cells allowed rapid growth in insect cells without an appreciable lag (Figure 7D, compare dashed and solid green lines). Thus, as anticipated, the rJ.03-G virus appears to behave identically to the parental JCV/03. In contrast, passage of rJ.03-S in C6/36 cells slightly improved replication in insect cells, but growth remained slower and less robust than that of the mosquito cell-derived viruses that encode G193 (Figure 7D, compare dashed orange line to dashed green and red lines).

**Figure 8:**
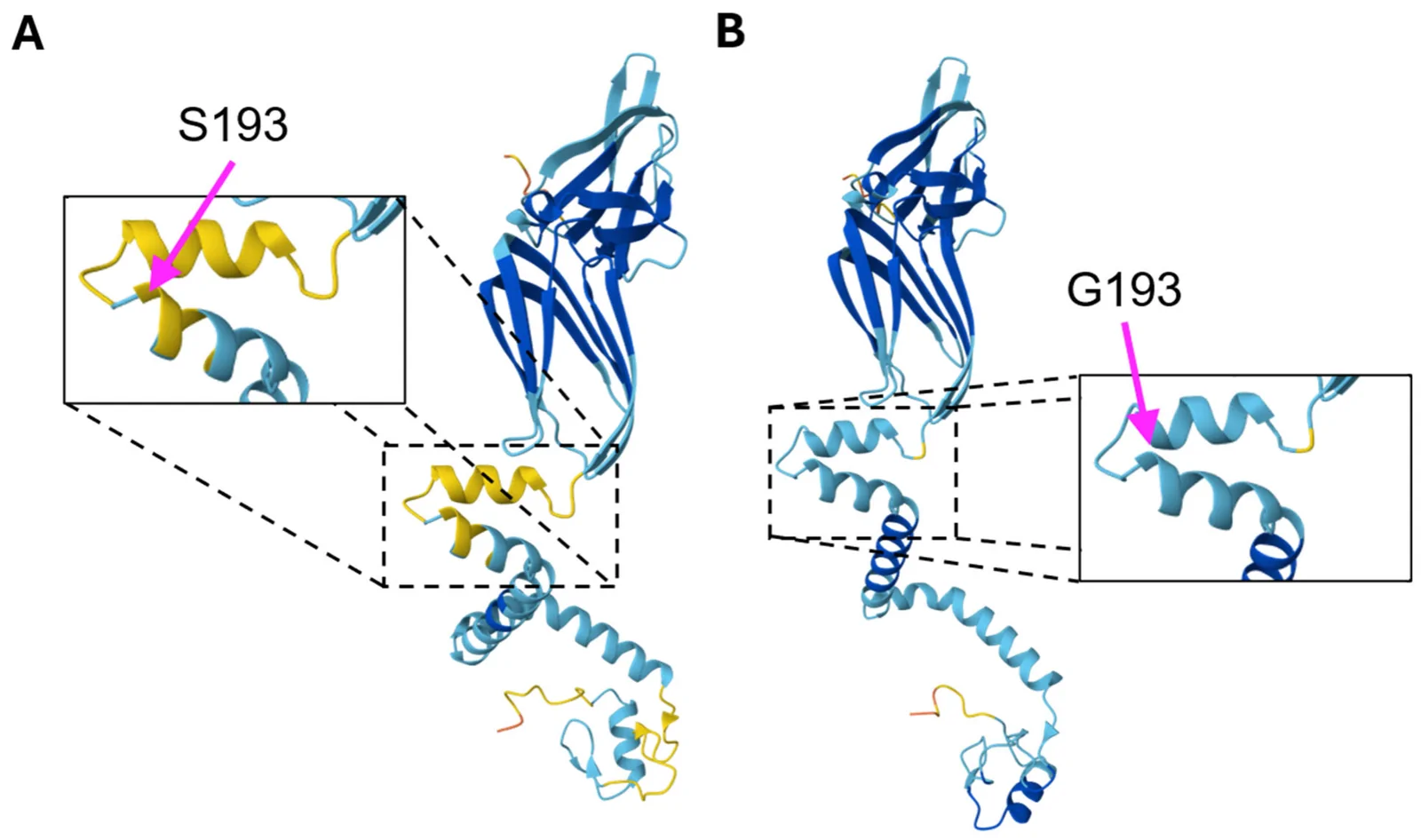
Predicted structures of JCV/03 Gn with residue 193 modeled as serine or glycine. AlphaFold-predicted structures of JCV/03 Gn, defined as residues 14 to 301 of the M segment polyprotein based on the coding-region boundaries described by Bennett et al. [32]. Models were generated from the JCV/03 M segment reference sequence with residue 193 specified as serine (**A**) or glycine (**B**). Models are colored by pLDDT, with dark blue indicating very high confidence (>90), light blue indicating confidence (70–90), yellow indicating low confidence (50–70), and orange indicating very low confidence (<50). Insets show enlarged views of the region surrounding residue 193. Fuchsia arrows indicate the position of residue 193.

To examine the potential structural consequences of variation at position 193, three-dimensional models of JCV/03 Gn containing either serine (Figure 8A) or glycine (Figure 8B) were generated. The AlphaFold 3-predicted structures were visually similar overall, with no substantial conformational differences apparent between the S193 and G193 models. However, local prediction confidence at position 193 was higher in the G193 model. The Cα predicted local distance difference test (pLDDT) score increased from 67.68 in the S193 model to 76.12 in the G193 model, corresponding to a shift from low confidence (yellow in Figure 8A) to confident (light blue in Figure 8B).

### 3.7. Multiple Independent M Segment Mutations Contribute to Enhanced Replication in C6/36 Cells

**Figure 9:**
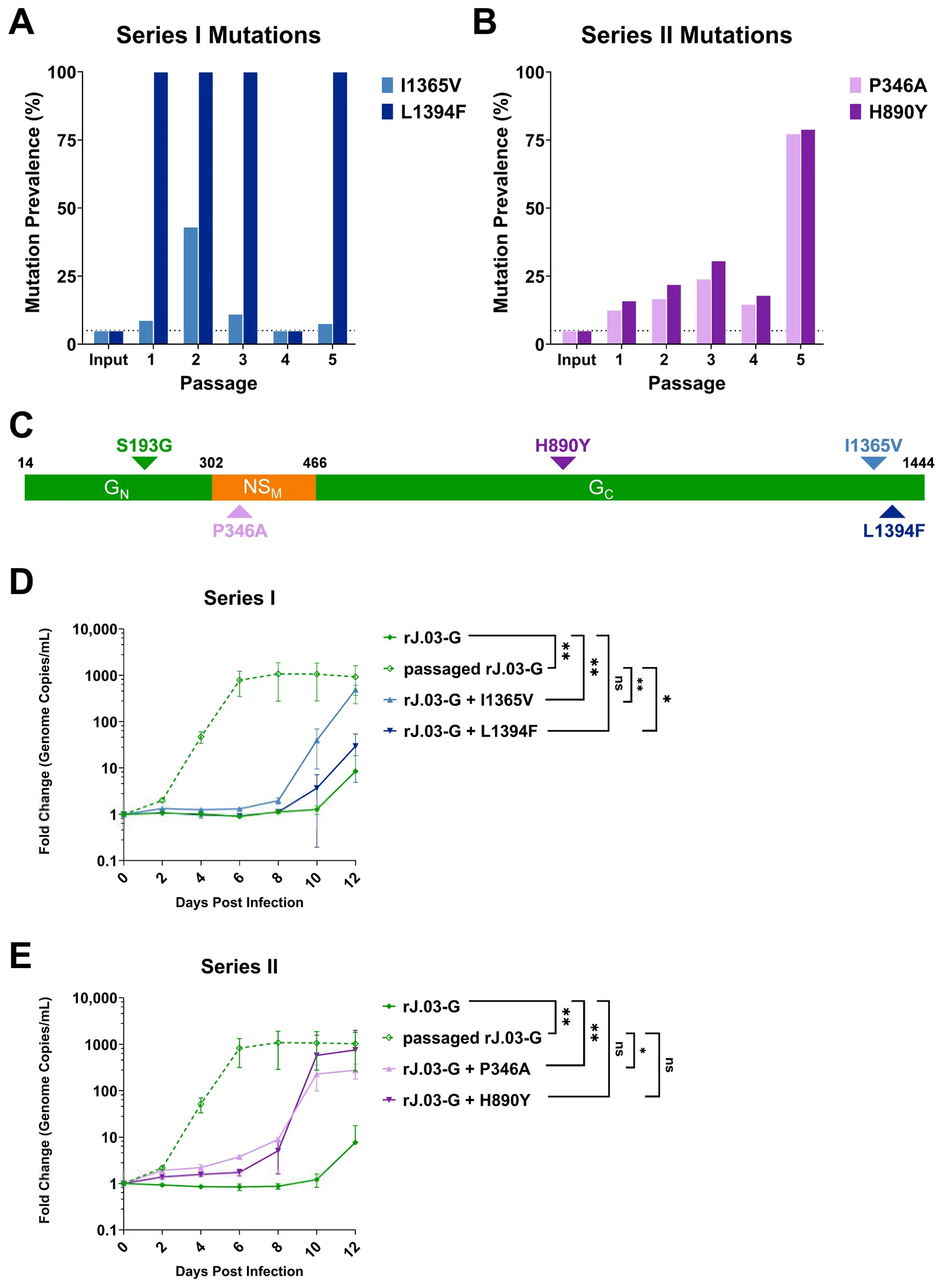
Multiple mutations across the M segment independently confer enhanced replication of JCV/03 in C6/36 cells. Mutation frequencies from Series I (**A**) and Series II (**B**) were determined by Illumina sequencing of viral RNA isolated at the final time point of each passage shown in Figure 6. Only mutations that rose appreciably above background variation and whose frequencies corresponded with the observed replication phenotypes are shown. The dotted line indicates the 5% variant-frequency detection cutoff. (**C**) Schematic of the JCV M segment polyprotein showing the Gn, NSm, and Gc regions and the positions of S193G and mutations identified during serial passage. Black numbers indicate amino acid positions within the complete glycoprotein precursor. (**D**,**E**) Multistep growth curves in C6/36 cells comparing rJ.03-G with recombinant viruses carrying individual M segment mutations identified in Series I (**D**) or Series II (**E**). Cells were infected with a target inoculum of 2 × 10^6^ genome copies, and viral RNA in supernatants collected at the indicated time points was quantified by qPCR. Titers are expressed as fold change in genome copies/mL relative to the corresponding input measured at 0 hpi. Measured inputs are provided in Supplemental Table S3. Data represent the mean ± SD of three replicate culture wells from one independent experiment. Fold-change values were log10-transformed, and area under the curve was calculated separately for each well across the full time course using a baseline of Y = 0. Comparisons were made using two-tailed paired t-tests, with wells matched by well number across conditions. *p* values are indicated as * *p* < 0.05, ** *p* < 0.01. ns—not significant.

In addition to the S193G substitution, sequencing identified numerous mutations that emerged during passage in C6/36 cells. All nonsynonymous mutations detected above the 5% cutoff are summarized in Supplemental Tables S7 and S8. Although the Series I and Series II experiments did not share any amino acid substitutions associated with adaptation, several M segment mutations displayed frequency changes that closely paralleled the replication phenotypes observed during the respective passage series. In Series I, two nonsynonymous mutations in Gc, I1365V and L1394F, increased in frequency during passage in C6/36 cells and subsequently declined following passage in Vero cells, consistent with the reversion phenotype apparent in the final passage (Figure 9A). In Series II, two unique M segment mutations, P346A and H890Y, were associated with efficient replication in C6/36 cells and persisted after passage in Vero cells, consistent with the stable adaptation phenotype observed in the series (Figure 9B). Figure 9C summarizes the positions of these mutations within the JCV M segment polyprotein.

To determine whether these mutations contributed directly to enhanced replication in insect cells, each mutation was introduced individually into the M segment of rJ.03-G. After launch and amplification in Vero cells, these recombinants were evaluated for growth in mosquito cells and compared to Vero cell-derived rJ.03-G and C6/36-derived rJ.03-G. As observed previously, Vero-derived rJ.03-G displayed a prolonged lag phase, with viral titers remaining near baseline until day 10 post-infection (Figure 9D,E, solid green lines). In contrast, C6/36-derived rJ.03-G transitioned to rapid replication by day 2 post-infection and reached peak titers by day 6 (Figure 9D,E, dashed green lines). The Series I Gc mutations produced subtle changes in replication kinetics. rJ.03-G + I1365V entered rapid replication at day 8 post-infection, approximately two days earlier than Vero-derived rJ.03-G and ultimately reached peak titers comparable to C6/36-derived rJ.03-G (Figure 9D, light blue line). rJ.03-G + L1394F also began increasing at day 8 post-infection, but peak titers only modestly exceeded those of Vero-derived rJ.03-G (Figure 9D, dark blue line). The Series II mutations produced a stronger phenotype. Recombinant viruses carrying the P346A or H890Y mutations displayed detectable replication prior to day 8 post-infection and then underwent rapid amplification beginning approximately day 8 (Figure 9E, purple lines). By day 10, both recombinants reached peak titers that were comparable to C6/36-derived rJ.03-G and remained stable through day 12. Thus, while all four mutations shortened the lag phase relative to Vero-derived rJ.03-G, they did not recreate the immediate, robust replication of C6/36-derived rJ.03-G.

In addition to P346A and H890Y, sequencing of Series II identified the NSm mutation W371R as a possible determinant of enhanced replication in C6/36 cells because its frequency increased during passages 1 and 2 in insect cells and subsequently declined during passage in Vero cells (Supplemental Table S8 and Supplemental Figure S3A). However, unlike P346A and H890Y, W371R was not detected in passage 5, suggesting it was not associated with the sustained adaptation phenotype observed in the growth curves. To directly test its contribution, recombinant rJ.03-G + W371R was generated and evaluated in C6/36 cells. The W371R-encoding virus exhibited an abbreviated lag phase relative to both parental JCV/03 and Vero-derived rJ.03-G. Whereas parental JCV/03 and rJ.03-G transitioned to rapid replication at approximately days 6 and 4 post-infection, respectively, rJ.03-G + W371R displayed exponential replication by day 2 post-infection (Supplemental Figure S3B). Despite this accelerated onset of replication, rJ.03-G + W371R reached peak titers approximately 10-fold lower than parental JCV/03 and Vero-derived rJ.03-G. Peak titers were comparable to C6/36-derived rJ.03-G, but they were reached approximately two days later. Therefore, although W371R enhanced replication kinetics in C6/36 cells, this change alone did not reproduce the fully adapted phenotype.

We also performed sequence analysis of JCV/03 derived from serial passage at lower cell density (Supplemental Figure S2). This analysis identified an M352V substitution in NSm as another potential determinant of enhanced replication in C6/36 cells (Supplemental Figure S4A). Recombinant rJ.03-G + M352V displayed an intermediate phenotype between Vero-derived and C6/36-derived rJ.03-G. Following an approximately 8-day lag phase, rJ.03-G + M352V underwent rapid replication and reached peak titers by day 12 post-infection (Supplemental Figure S4B). Peak titers were comparable to those of C6/36-derived rJ.03-G and greater than ten-fold higher than Vero-derived rJ.03-G. However, unlike C6/36-derived rJ.03-G, which entered rapid replication by day 2 and peaked by day 6, the M352V mutant retained a substantial replication delay. Thus, similar to the other M segment mutations evaluated in this study, M352V partially enhanced replication in C6/36 cells but was insufficient to fully reproduce the adapted phenotype.

Together, these data demonstrate that multiple independent mutations within the M segment can act to enhance JCV/03 replication in C6/36 cells. However, no single mutation fully recapitulated the rapid replication kinetics of C6/36-derived virus, suggesting that efficient adaptation likely reflects the combined effects of multiple mutations.

## 4. Discussion

Jamestown Canyon virus (JCV) is unusual among arboviruses in its broad host and vector range, having been detected in 46 mosquito species, several tabanids, and a variety of mammalian hosts [7,8,12,14,15,16,17,18,19,40]. To investigate the viral determinants that may contribute to this ecological flexibility, we compared the prototype strain JCV/61 with the more contemporary strain JCV/03. Previous work demonstrated that, despite their substantial genetic similarity, these strains elicited distinct outcomes in vivo. In *Aedes albopictus*-derived C6/36 cells, JCV/03 displayed a prolonged lag phase followed by an abrupt increase in replication, whereas JCV/61 replicated immediately after infection. Importantly, a single passage of JCV/03 in C6/36 cells consistently eliminated the lag phase and conferred a heritable enhancement of replication in mosquito cells. Sequencing of the resulting viral populations identified mutations distributed across all three proteins encoded by the M segment that increased in frequency during infection of mosquito cells. Using reverse genetics to evaluate the effects of individual mutations on replication in C6/36 cells, we found that no single mutation fully recapitulated the adapted phenotype. Collectively, these findings support a model in which efficient replication in mosquito cells can arise through multiple evolutionary pathways.

Viral genotype was not the only factor influencing the JCV/03 lag-burst replication phenotype; C6/36 culture conditions also shaped the overall course of infection. Notably, we determined that increasing the initial plating density of the C6/36 cells dramatically shortened the lag phase and increased the magnitude of the replication burst. It may be that higher cell densities permitted more efficient cell–cell spread, thereby shortening the replication delay. Another possibility results from the heterogeneous nature of C6/36 cells. Some subpopulation of cells may differ in permissiveness to JCV/03 replication and under this model, viral replication remains below the limit of detection until a sufficient number of permissive cells become available. More broadly, it remains unclear whether the lag-burst phenotype is unique to C6/36 cells or reflects a characteristic of mosquito-derived cultures. Future studies in additional insect cell lines are necessary to address this question and evaluate alternative explanations for the phenotype.

A longstanding theory in arbovirus evolution is that alternating replication between vertebrate and arthropod hosts constrains viral evolution [41,42,43]. Under this trade-off hypothesis, mutations that improve fitness in one host are expected to reduce fitness in the alternate host, thereby limiting specialization and slowing long-term evolution. Our findings are consistent with a more nuanced view of arbovirus evolution in which host alternation constrains some outcomes without completely preventing evolutionary innovation. In the first serial passage experiment (Figure 6A), adaptation to C6/36 cells produced little detectable effect on replication in Vero cells, yet the phenotype reverted following mammalian passage, suggesting that the adaptive changes were not stably maintained in the absence of mosquito cell-specific selection. In contrast, the second serial passage experiment (Figure 6B) demonstrated that adaptation acquired in mosquito cells could persist after passage through mammalian cells, although this persistence was accompanied by reduced replication in Vero cells. Collectively, these outcomes suggest that alternating-host replication acts not as an absolute evolutionary barrier but rather as a selective filter that may favor adaptive solutions compatible with both hosts.

A strength of this study was the development and application of a plasmid-based reverse genetics system for JCV/03. Concurrent efforts by Alyssa Evans and colleagues established a complementary reverse genetics platform for JCV/61 that has proven valuable for large-scale segment reassortment studies [44,45]. In contrast, the system described here was particularly well suited for targeted mutagenesis, allowing direct evaluation of specific amino acid substitutions. Together, these systems provide a foundation for future studies examining both segment-level and residue-level determinants of JCV host adaptation.

Residue 193 in the Gn coding region was identified as a difference between our JCV/03 virus stocks and the published reference JCV/03 sequence. However, analysis of this discrepancy revealed that the glycine encoded in our stocks likely represents the wild-type sequence, whereas the serine reported in the reference sequence is likely due to a sequencing error. This conclusion is supported by several lines of evidence. First, all available JCV sequences, including JCV/61, encode glycine at position 193. Second, recombinant JCV/03 encoding serine at position 193 replicates poorly in both mammalian and insect cells. Finally, a recombinant JCV/03 carrying glycine at position 193 displayed replication kinetics identical to our JCV/03 stocks, including the lag-burst phenotype in C6/36 cells. JCV/61 and JCV/03 are therefore identical at residue 193 despite their markedly different replication kinetics in C6/36 cells. The differences in replication in mosquito cells for these two strains likely arise from the numerous amino acid substitutions distributed throughout the M segment.

Using targeted mutagenesis, we evaluated S193G together with six M-segment mutations identified during serial passage: P346A, M352V, and W371R in NSm and H890Y, I1365V, and L1394F in Gc. Each of the six passage-associated mutations enhanced replication in C6/36 cells when introduced individually into rJ.03-G. The location and identity of each mutation provide clues to their potential mechanism of action. Based on structural mapping of La Crosse virus glycoproteins, H890Y localizes to stalk subdomain 2 of Gc [46]. Mutants lacking the stalk and head domains retained the ability to undergo low pH-triggered fusion, suggesting this region may serve a structural rather than pH-sensing role [47,48]. Substitution of histidine with tyrosine introduces a hydroxyl-containing aromatic side chain capable of altering hydrogen bonding, potentially influencing protein conformation or interactions. Neither the location nor the biochemical properties of I1365V provide an obvious explanation for how this substitution enhances replication in C6/36 cells. However, because the mutation increased during passage in mosquito cells and declined following passage in Vero cells (Supplemental Table S7), the selective advantage conferred by I1365V appears to be host dependent. V1365 appears in nearly 30 orthobunyavirus sequences in the BV-BRC and is the most common residue at this position after I1365. Likewise, L1394F was not unique to our study. Among sequences on the BV-BRC, leucine and phenylalanine were both common at this position. Further investigation is needed to determine whether viruses carrying V1365 and F1394 were recovered from mosquito or mammalian hosts.

Structurally, L1394F localizes immediately adjacent to a predicted transmembrane domain in a region analogous to the membrane-proximal external region (MPER) found in other viral fusion proteins [49,50,51]. Studies have shown that residues within the MPER contribute to viral entry, intracellular trafficking, and membrane fusion, suggesting this region plays an important structural role during infection [52,53,54]. Interestingly, although L1394F repeatedly reached fixation during serial passage, recombinant rJ.03-G + I1365V produced a more pronounced enhancement than rJ.03-G + L1394F (Figure 9D). This discrepancy suggests that mutation frequency alone may not directly reflect the magnitude of phenotypic contribution. Notably, I1365V and L1394F emerged together during serial passage, raising the possibility that these substitutions function cooperatively and that both may be required to fully reproduce the adapted phenotype. Determining the mechanistic basis for these phenotypes will require direct analysis of how individual and combined mutations affect glycoprotein fusogenicity and membrane interactions.

Three of the mutations identified and tested mapped to NSm, one of the least well-characterized proteins encoded by orthobunyaviruses. This finding was particularly notable because, with the exception of the N-terminal region, NSm is largely dispensable for replication in cultured mammalian and arthropod cells [28,29,30,55]. P346A and M352V localized to NSm domain II, a predicted luminal domain. When engineered into rJ.03-G, both P346A and M352V enhanced JCV/03 replication in C6/36 cells, demonstrating that changes within this region can improve viral replication without disrupting NSm function. Both positions are also highly conserved and invariant among California serogroup orthobunyaviruses, including La Crosse, California encephalitis, Snowshoe Hare, and Tahyna viruses. The final NSm mutation, W371R, localized to domain III, a canonical transmembrane domain implicated in membrane association and efficient viral replication [55,56]. For Bunyamwera virus, deletion of domain III substantially impairs replication [48]. However, rather than impairing replication, W371R markedly accelerated JCV/03 replication in C6/36 cells (Supplemental Figure S3). Thus, the repeated emergence of NSm mutations during serial passage, together with their ability to enhance replication when tested individually, supports a role for NSm in mosquito cell adaptation, although the mechanism underlying these effects remains unclear.

Several questions remain regarding the biological relevance of these findings. Much of this study was conducted in relatively immunodeficient cell lines, as C6/36 cells lack a functional antiviral RNAi response and Vero cells cannot produce type I interferons [57,58]. It also remains unclear whether the JCV/03 lag-burst phenotype, its rapid adaptation, and the effects of the associated M-segment mutations are specific to C6/36 cells or extend more broadly across mosquito-derived systems. Furthermore, because no individual mutation fully recapitulated the phenotype acquired during serial passage, combinations of mutations may be required to reproduce the rapid replication kinetics of mosquito cell-adapted virus. Studies in additional mosquito cell lines, particularly those with intact antiviral responses, and in live mosquitoes will be required to determine whether these findings are more generally applicable.

Interpretation of these findings must also account for several limitations of the experimental conditions and methods used. C6/36 cells were maintained in L-15 medium with supplemental CO_2_, potentially reducing the pH of the culture medium. Supplementation with 25 mM HEPES to limit this decline shortened the JCV/03 replication lag in mosquito cells, demonstrating that replication was sensitive to pH. In our experience, supplemental CO_2_ lowered the pH of L-15 medium by approximately 0.3 units, but it remains unclear whether this reduction was sufficient to influence JCV/03 adaptation. The standard culture requirements of C6/36 and Vero cells also necessitated their maintenance at different temperatures. Therefore, the selective pressure from cell type cannot be fully distinguished from that of pH, temperature, or other culture-specific conditions. Nevertheless, these considerations do not alter the central observation that JCV/03 acquired a heritable enhancement of replication after a single passage in C6/36 cells, whereas JCV/61 replication remained comparatively unchanged.

Finally, with the exception of the experiments shown in Figure 1 and Figure 3, inocula were normalized by viral genome copy number, and viral output was quantified by qPCR rather than by plaque assay. This approach avoided potential host cell-dependent bias associated with titrating all viruses in Vero cells, particularly if adaptation to C6/36 cells reduced plaque-forming efficiency in mammalian cells. However, because genome copy-to-PFU ratios differed among viruses and stocks, genome-normalized inocula did not always represent equivalent infectious MOIs. Moreover, defective viral genomes can accumulate during serial passage, particularly at high MOI, and qPCR captures any genome regardless of whether it is associated with an infectious particle. Consequently, an increase in genome copies may not correspond to an equivalent increase in infectious virus. Expressing viral output as fold change relative to the genome-copy input normalized the results to the amount of detectable viral genome added but did not eliminate this limitation or determine the contribution of defective particles. Nevertheless, experiments incorporating PFU-normalized inocula or plaque-assay quantification reproduced the same relative replication phenotypes, indicating that the principal findings were not solely attributable to genome-based normalization or quantification.

In summary, this study identifies a previously unrecognized mosquito cell-specific replication phenotype in a Jamestown Canyon virus isolate and demonstrates that adaptation to mosquito cells can occur rapidly through multiple independent mutations within the M segment. The discovery that Gn, Gc, and NSm mutations enhance replication highlights the diversity of genetic pathways available for mosquito cell adaptation. More broadly, these findings establish a flexible reverse genetics system for JCV/03 and provide a framework for dissecting the viral determinants that govern host-specific fitness and arbovirus evolution.

## Data Availability

All data necessary to evaluate the conclusions of this study are included in the article and supplemental materials. The JCV/03 M-segment sequence generated in this study has been deposited in GenBank under accession number PZ903552. Additional data and reagents are available from the corresponding author upon request.

## Supplementary Materials

The following supporting information can be downloaded at: https://www.mdpi.com/article/10.3390/v18091018/s1, Figure S1: Parental, uncloned JCV/03 and biological clones of JCV/03 display the lag-burst growth phenotype in C6/36 cells; Figure S2: Initial plating density does not alter the long-term outcome of serial passage; Figure S3: W371R enhances replication kinetics of rJ.03-G in C6/36 cells; Figure S4: M352V enhances replication kinetics of rJ.03-G in C6/36 cells; Table S1: Primers used for sequencing biological clones and verifying point mutations; Table S2: Sequence comparison between prototype JCV clones and the designated reference sequence; Table S3: qPCR-quantified input genome copies used as baseline values for fold-change; Table S4: Primers used for constructing JCV/03-pN expression plasmid; Table S5: qPCR primers [59]; Table S6: Primers used to introduce point mutations in the JCV/03 pM launch plasmid; Table S7: Combined sequencing data for all non-synonymous mutations discovered in Series I; Table S8: Combined sequencing data for all non-synonymous mutations discovered in Series II.
