# Supplementary material for: Jamestown Canyon Virus Rapidly Adapts to Mosquito Cells Through Multiple M Segment Mutations
Source: Viruses. 2026 Sep 15;18(9):1018. doi: 10.3390/v18091018 (PMC13611844; doi:10.3390/v18091018)
Supplement: Supplementary file 1 [file viruses-18-01018-s001.zip › viruses-4414650-supplementary.pdf]

Supplemental Materials

Supplemental Table S1. Primers used for sequencing biological clones and verifying point mutations

Target	Forward Primer (5'-3')	Reverse Primer (5'-3')
JCV Small Segment [31]	TGACCAGTAGTGTACTCCAC	CAAGCAGTAGTGTGCTCCAC
JCV Medium Segment	AGTAGTGTGCTACCAAGTATATTTAAATGATTC	AGTAGTGTACTACCAAGTATAGAAAACGTTC
JCV Large Segment	AGTAGTGTGCTCCTATTTAC	AGTAGTGTACTCCTATTTACAAAACTTAC

Supplemental Table S2. Sequence comparison between prototype JCV clones and the designated reference sequence. Sequences derived from the prototype strain of JCV/61 and JCV/03 were aligned to the corresponding reference genome. JCV/61 (Small: U12796.1, Medium: U88058.1, Large: NC_043559.1). JCV/03 (Small: HM007353.1, Medium: HM007354.1, Large: HM007355.1) * represents a mutation introduced by the primer used for sequencing. N/D – not determined at this time. N/A – not available because the mutation occurred in a non-coding region and therefore, there was no associated amino acid.

Virus & Segment	Nucleotide			Amino Acid		
	Position	Reference	Sequencing	Position	Reference	Sequencing
JCV/61 S	9	A	G*	N/A		
JCV/61 M	1-25		N/D			
JCV/61 M	328	T	C	92	N	N
JCV/61 M	759	C	T	236	T	I
JCV/61 M	885	A	C	278	N	T
JCV/61 M	1067	C	G	339	L	V
JCV/61 M	1140	C	V	363	T	I
JCV/61 M	1174	T	A	374	F	L
JCV/61 M	1176-1177	GC	TG	375	C	L
JCV/61 M	1240	G	A	396	A	A
JCV/61 M	1534	C	T	494	T	T
JCV/61 M	1619	G	A	523	G	R
JCV/61 M	1738	C	T	562	A	A
JCV/61 M	1783	A	C	577	K	N
JCV/61 M	1846	T	C	598	V	V
JCV/61 M	2051	G	A	667	E	K
JCV/61 M	2466	G	A	805	G	D
JCV/61 M	3130	G	A	1026	K	K
JCV/61 M	3190	A	G	1046	G	G
JCV/61 M	3852	G	A	1267	S	N
JCV/61 M	3931	G	A	1293	E	E
JCV/61 M	4070	C	A	1340	H	N
JCV/61 M	4485-4510		N/D			
JCV/61 L	1-26		N/D			
JCV/61 L	5013	A	G	1652	L	L
JCV/61 L	6631	G	A	2192	D	N
JCV/61 L	6900-6960		N/D			
JCV/03 S	9	A	G*			
JCV/03 M	1-24		N/D			
JCV/03 M	629	A	G	193	S	G
JCV/03 M	4484-4510		N/D			
JCV/03 L	1-22		N/D			
JCV/03 L	6935-6957		N/D			

Supplemental Table S3. qPCR-quantified input genome copies used as baseline values for fold-change.
For experiments titrated by qPCR, an aliquot of each inoculum was retained at 0 hpi, and genome copies were quantified by qPCR. The measured genome copies per well, rather than the intended inoculum, were used as the baseline for calculating fold change in the indicated experiments. Figure numbers beginning with “S” refer to supplemental figures. A blank entry in the Panel column indicates that the corresponding figure contains only one panel and therefore has no letter or number designation. The Virus column identifies the virus used, except for Figure 4, in which all experiments used JCV/03 and the entries distinguish the medium conditions. Alternating shading distinguishes groups of rows associated with different figures or panels.

Figure	Panel	Virus	Replicate Well #	Genome Copies
2	A	JCV/61		1.55E+07
2	A	JCV/03		9.30E+06
3		JCV/61		7.00E+06
3		JCV/03		5.36E+05
4		WT	1	1.93E+06
4		WT	2	9.73E+05
4		Buffered	1	1.12E+06
4		Buffered	2	2.07E+06
4		Conditioned batch A		1.28E+06
4		Conditioned batch B		1.07E+06
4		Conditioned batch C		1.57E+06
5		JCV/61		5.35E+07
5		JCV/61 Reinfection: high density		1.16E+07
5		JCV/61 Reinfection: low density		1.20E+07
5		JCV/03		9.98E+06
5		JCV/03 Reinfection: high density		2.38E+06
5		JCV/03 Reinfection: low density		3.92E+06
6	A1	JCV/03		7.21E+05
6	A2	JCV/03		5.24E+05
6	A2	passaged JCV/03		1.07E+06
6	A3	JCV/03		3.34E+05
6	A3	passaged JCV/03		3.06E+05
6	A4	JCV/03		3.58E+05
6	A4	passaged JCV/03		3.26E+05
6	A5	JCV/03		9.73E+05
6	A5	passaged JCV/03		5.00E+06
6	B1	JCV/03		5.36E+05
6	B2	JCV/03		9.98E+06
6	B2	passaged JCV/03		2.38E+06

Figure	Panel	Virus	Replicate Well #	Genome Copies
6	B3	JCV/03		2.25E+06
6	B3	passaged JCV/03		2.40E+06
6	B4	JCV/03		1.83E+05
6	B4	passaged JCV/03		1.74E+05
6	B5	JCV/03		1.93E+06
6	B5	passaged JCV/03		2.44E+06
7	B	JCV/03	1	6.35E+05
7	B	JCV/03	2	7.20E+05
7	B	JCV/03	3	8.22E+05
7	B	rJ.03-S	1	7.70E+05
7	B	rJ.03-S	2	7.43E+05
7	B	rJ.03-S	3	8.97E+05
7	B	rJ.03-G	1	8.16E+05
7	B	rJ.03-G	2	8.42E+05
7	B	rJ.03-G	3	7.12E+05
7	C	JCV/03		2.60E+06
7	C	rJ.03-S		1.10E+06
7	C	rJ.03-G		2.73E+06
7	D	JCV/03		9.83E+06
7	D	passaged JCV/03	1	3.76E+07
7	D	passaged JCV/03	2	1.24E+07
7	D	passaged JCV/03	3	6.26E+06
7	D	rJ.03-G		9.37E+06
7	D	passaged rJ.03-G	1	8.29E+06
7	D	passaged rJ.03-G	2	5.87E+06
7	D	passaged rJ.03-G	3	7.62E+06
7	D	passaged rJ.03-S	1	4.24E+06
7	D	passaged rJ.03-S	2	8.27E+06
7	D	passaged rJ.03-S	3	5.22E+06
9	D	rJ.03-G		4.01E+06
9	D	passaged rJ.03-G	1	9.16E+05
9	D	passaged rJ.03-G	2	6.19E+05
9	D	passaged rJ.03-G	3	7.39E+05
9	D	rJ.03-G + I1365V		1.05E+06
9	D	rJ.03-G + L1394F		1.20E+06
9	E	rJ.03-G		3.87E+06
9	E	passaged rJ.03-G	1	7.57E+05
9	E	passaged rJ.03-G	2	4.72E+05
9	E	passaged rJ.03-G	3	5.99E+05
9	E	rJ.03-G + P346A		7.52E+05
9	E	rJ.03-G + H890Y		9.24E+05
S1		Parental JCV/03		5.53E+05
S1		JCV/03 Clone A		4.66E+05

Figure	Panel	Virus	Replicate Well #	Genome Copies
S1		JCV/03 Clone B		3.24E+06
S1		JCV/03 Clone C		7.35E+05
S1		JCV/03 Clone D		1.65E+06
S2	1	JCV/03		5.36E+05
S2	2	JCV/03		9.98E+06
S2	2	passaged JCV/03 high density		2.38E+06
S2	2	passaged JCV/03 low density		3.92E+06
S2	3	JCV/03		2.25E+06
S2	3	passaged JCV/03 high density		2.40E+06
S2	3	passaged JCV/03 low density		2.71E+06
S2	4	JCV/03		1.83E+05
S2	4	passaged JCV/03 high density		1.74E+05
S2	4	passaged JCV/03 low density		1.76E+05
S2	5	JCV/03		1.93E+06
S2	5	passaged JCV/03 high density		2.44E+06
S2	5	passaged JCV/03 low density		5.29E+06
S3	B	JCV/03		9.83E+06
S3	B	rJ.03-G		9.37E+06
S3	B	passaged rJ.03-G	1	8.29E+06
S3	B	passaged rJ.03-G	2	5.87E+06
S3	B	passaged rJ.03-G	3	7.62E+06
S3	B	rJ.03-G + W371R		8.80E+06
S4	B	rJ.03-G		3.87E+06
S4	B	passaged rJ.03-G	1	7.57E+05
S4	B	passaged rJ.03-G	2	4.72E+05
S4	B	passaged rJ.03-G	3	5.99E+05
S4	B	rJ.03-G + M352V		7.83E+05

Supplemental Table S4. Primers used for constructing JCV/03-pN expression plasmid

Target	Forward Primer (5'-3')	Reverse Primer (5'-3')
JCV/03 N	AAACACGATGATAATATGGGAGATT TGGTTTTCTATGATG	GATCTCAGTGGTCTATCAAGGCAG TTTTACACCG
pIRES-polyA vector	TAGACCACTGAGATCTGACTGAAA	ATTATCATCGTGTTTTTCAAAGGAA

Supplemental Table S5. qPCR primers

Target	Forward Primer (5'-3')	Reverse Primer (5'-3')
JCV N	CCTGCCACTCTCCAAACTTAG	TTCCTTAATGCAGCAAAAGCC
C6/36 Actin [59]	CACGAACTGGGACGATATGGA	CGGTTAGCCTTCGGGTTCAG

Supplemental Table S6. Primers used to introduce point mutations in the JCV/03 pM launch plasmid.

Mutation	Forward Primer (5'-3')	Reverse Primer (5'-3')
S193G	TACTCCCTGGTTCAATAGCTAACTC TATATGCCA	TTGAACCAGGGAGTATTGTTCTGTG TAAAAAC
P346A	TGATTTAGCCGATGATATGGTACAA ATGGC	GTACCATATCATCGGCTAAATCATCT AGAGTG
M352V	GATATGGTACAAGTGGCTGAGAGA GTGAACATTTAC	CACTCTCTCAGCCACTTGTACCATA TCATC
W371R	GTGTTACACGGGCTTTCTTGCTAC TTG	AAGCCCGTGTAACACCATAATTTATA ATGAT
H890Y	ATGTGGGATATTTTTGCCTTAGTCC CAGATGC	AAAAATATCCCACATCTTGATCTTTA TCATAA
I1365V	CTGCATATGTCAAAGAGAAAGATG AGAGATGT	CTTTGACATATGCAGTTTGGTCTAC TGGTGCA
L1394F	CCTTTTAAAAATTTTTTCGGCTCTT ATATTGGTATATTTTATACAGGC	GCCGAAAAAATTTTTAAAAGGCTCT AATATAACTTGAAATCC

Supplemental Table S7. Combined sequencing data for all non-synonymous mutations discovered in Series I.
Mutation frequencies from Series I were determined by Illumina sequencing of viral RNA isolated at the final time point of each passage. Mutations highlighted in green were chosen for follow-up in Figure 9. Amino acid numbering was based on the open reading frame in the reference genome for the polymerase (large segment), polyprotein (medium segment), and nucleocapsid (small segment). Accession numbers for JCV/03, Small: HM007353.1, Medium: HM007354.1, Large: HM007355.1. Mutation S193G is not shown. Minimum variant frequency cutoff of 5%.

Gene	Mutation	Input	Pass 1- C6/36	Pass 2- C6/36	Pass 3- Vero	Pass 4- Vero	Pass 5- C6/36
Polymerase	A1504T		17.7%	15.0%	12.3%	5.3%	
Polymerase	F1962L				7.0%	7.0%	14.0%
NSm	D347N		13.7%		7.4%	5.6%	
NSm	L378P				6.4%	50.8%	
NSm	Y395H		44.3%	44.3%	70.5%	38.0%	66.0%
NSm	M404V		6.3%				
NSm/Gc	K467E			17.0%			
Gc	D514N					38.6%	
Gc	R590W						8.9%
Gc	E625G	24.7%	25.4%	5.7%	16.1%	53.4%	11.1%
Gc	H890L		9.3%	14.6%			
Gc	T1233I						73.1%
Gc	P1352S	23.1%					
Gc	I1365V		8.8%	43.1%	11.1%		7.6%
Gc	1393_1394insF		9.4%	15.6%	15.8%	7.7%	17.0%
Gc	L1394F		100.0%	100.0%	100.0%		100.0%
Gc	F1402Y		47.2%	41.8%	68.4%	42.0%	68.0%

Supplemental Table S8. Combined sequencing data for all non-synonymous mutations discovered in Series II.
Mutation frequencies from Series II determined by Illumina sequencing of viral RNA isolated at the final time point of each passage. Mutations highlighted in green were chosen for follow-up in Figure 9. Amino acid numbering based on the open reading frame in the reference genome for the polymerase (large segment), polyprotein (medium segment), and nucleocapsid (small segment). Accession numbers for JCV/03, Small: HM007353.1, Medium: HM007354.1, Large: HM007355.1. Mutation S193G is not shown. Minimum variant frequency cutoff of 5%.

Gene	Mutation	Input	Pass 1- C6/36	Pass 2- C6/36	Pass 3- Vero	Pass 4- Vero	Pass 5- C6/36
Polymerase	V1656I						6.6%
Polymerase	I1706T			8.2%			
Gn	A174V			7.9%			
NSm	L342P		9.8%			7.3%	
NSm	P346A		12.6%	16.8%	24.0%	14.7%	77.4%
NSm	W371R		51.2%	74.9%	12.3%		
NSm	G377D		7.0%				
NSm	C402Y		11.7%		14.8%	6.7%	
Gc	I540L				32.7%	62.1%	17.2%
Gc	H554R					6.7%	
Gc	E625G	24.7%	10.5%			6.4%	
Gc	T628I						34.7%
Gc	H890Y		16.0%	22.0%	30.7%	18.0%	79.0%
Gc	H890L						
Gc	T1233I						15.6%
Gc	P1352S	23.1%					
Gc	Y1364H			71.2%			
Gc	S1397F						63.9%
Gc	G1400S				5.8%	9.1%	9.4%
N/NSs	G22G/ G16V					7.9%	

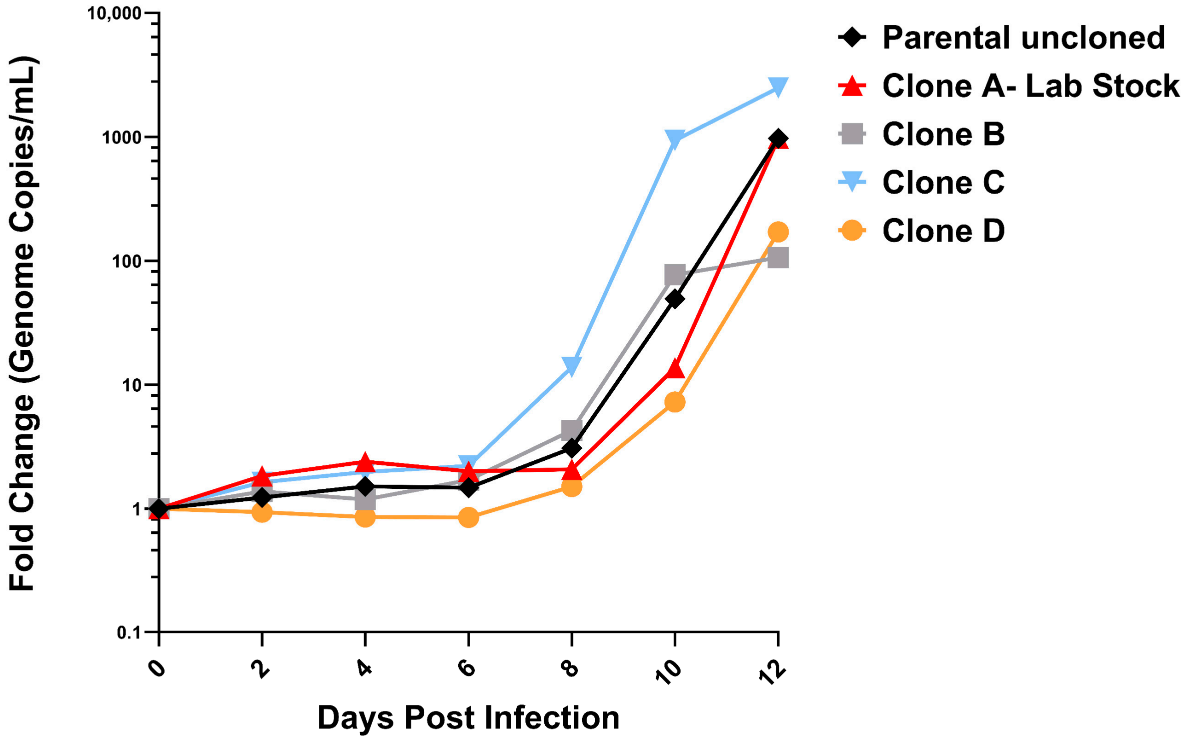

Supplemental Figure S1. Parental, uncloned JCV/03 and biological clones of JCV/03 display the lag-burst growth phenotype in C6/36 cells.
C6/36 cells were infected with parental, uncloned JCV/03 or one of four biological clones obtained after three rounds of terminal dilution using a target MOI of 0.003. Clone A was the laboratory stock used throughout this study. Supernatants were collected at the indicated time points and viral RNA was quantified by qPCR to determine genome copies per mL. Values are expressed as fold change in genome copies per mL relative to the corresponding input measured by qPCR at 0 hpi. Measured input values are provided in Supplemental Table S3. A single culture well was used for each virus, with one supernatant sample collected at each time point. Each culture sample was measured in technical triplicate by qPCR.

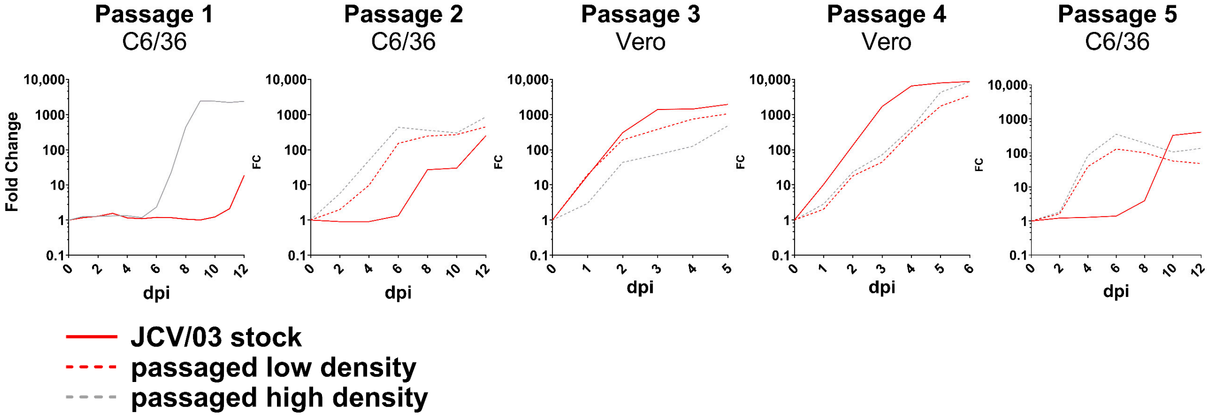

Supplemental Figure S2. Initial plating density does not alter the long-term outcome of serial passage. A five-passage series was performed using low-density C6/36 cultures during Passage 1, followed by the passage scheme described for Figure 6. For Passage 1, C6/36 cells were plated at a density six-fold lower than the high-density condition used for the passage series shown in Figure 6. JCV/03 was passaged twice in C6/36 cells (Passages 1 and 2), followed by two passages in Vero cells (Passages 3 and 4), and subsequently returned to C6/36 cells for Passage 5. C6/36 or Vero cells were infected with parental JCV/03 (solid red line), JCV/03 from the low-density passage series (dashed red line), or JCV/03 from the high-density passage series shown in Figure 6 (dashed gray line) using a target inoculum of 2×10^6 genome copies. Supernatants were collected every other day during C6/36 time courses and daily during Vero time courses. A single culture well was used for each virus in each passage, with one supernatant sample collected at each time point. Viral RNA was quantified by qPCR to determine genome copies per mL. Values are expressed as fold change (FC) in genome copies per mL relative to the corresponding input measured by qPCR at 0 hpi. Measured input values are provided in Supplemental Table S3. Each culture sample was measured in technical triplicate by qPCR.

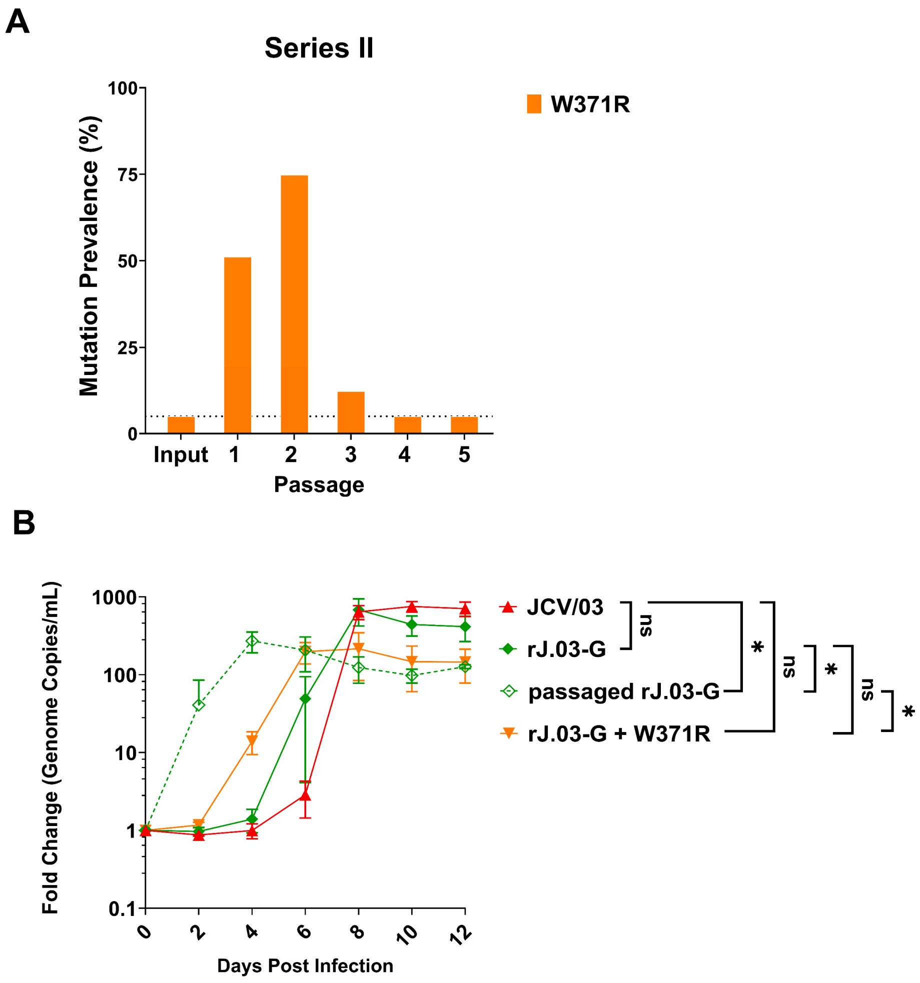

Supplemental Figure S3. W371R enhances replication kinetics of rJ.03-G in C6/36 cells. (A) Frequency of W371R during Series II determined by Illumina sequencing of viral RNA isolated at the final time point of each passage. The dotted line indicates the 5% variant-frequency detection cutoff. (B) Multistep growth curves comparing JCV/03, rJ.03-G, C6/36-passaged rJ.03-G, and rJ.03-G + W371R in C6/36 cells. Cells were infected with a target inoculum of 2×10^6 genome copies, and viral RNA in supernatants collected at the indicated time points was quantified by qPCR. Titers are expressed as fold change in genome copies/mL relative to the corresponding input measured at 0 hpi. Measured inputs are provided in Supplemental Table S3. Data represent the mean ± SD of three replicate culture wells from one independent experiment. Each sample was quantified in technical triplicate, and technical replicates were averaged before analysis. Fold-change values were log10-transformed, and area under the curve (AUC) was calculated separately for each well across the full time course with a baseline of Y = 0. AUC values were compared using two-tailed paired t-tests, with wells matched by well number across conditions. Statistical significance is indicated as *$P < 0.05$. ns – not significant.

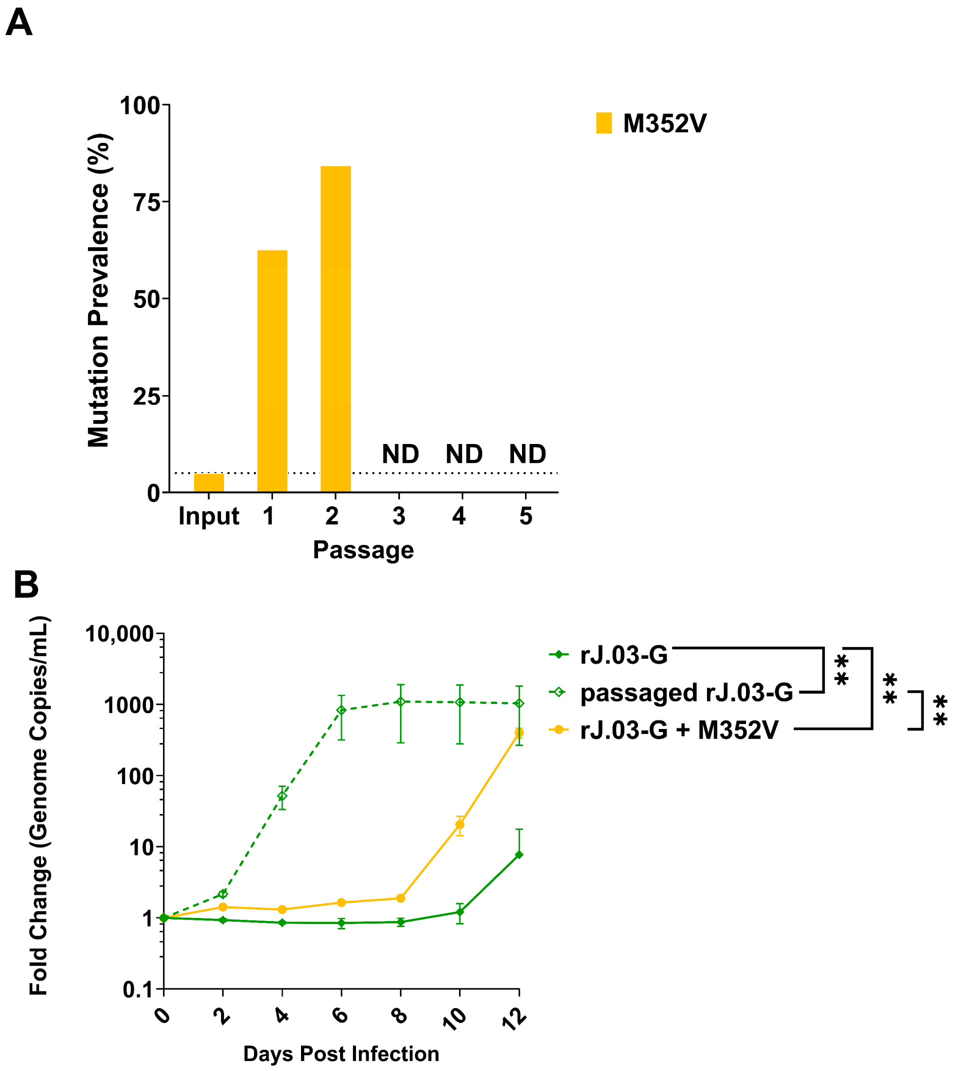

Supplemental Figure S4. M352V enhances replication kinetics of rJ.03-G in C6/36 cells. (A) Frequency of M352V during serial passage determined by Illumina sequencing of viral RNA isolated at the final time point of each passage. ND indicates not determined. The dotted line indicates the 5% variant-frequency detection cutoff. (B) Multistep growth curves comparing Vero-derived rJ.03-G, C6/36-passaged rJ.03-G, and rJ.03-G + M352V in C6/36 cells. Cells were infected with a target inoculum of 2×10^6 genome copies, and viral RNA in supernatants collected at the indicated time points was quantified by qPCR. Titers are expressed as fold change in genome copies/mL relative to the corresponding input measured at 0 hpi. Measured inputs are provided in Supplemental Table S3. Data represent the mean ± SD of three replicate culture wells from one independent experiment. Each sample was quantified in technical triplicate, and technical replicates were averaged before analysis. Fold-change values were log10-transformed, and area under the curve (AUC) was calculated separately for each well across the full time course with a baseline of Y = 0. AUC values were compared using two-tailed paired t-tests, with wells matched by well number across conditions. Statistical significance is indicated as $^{**}P < 0.01$.
